# Systematically Constructing Kinetic Transition Network in Polypeptide from Top to Down: Trajectory Mapping

**DOI:** 10.1371/journal.pone.0125932

**Published:** 2015-05-11

**Authors:** Linchen Gong, Xin Zhou, Zhongcan Ouyang

**Affiliations:** 1 Institute for Advanced Study, Tsinghua University, Beijing 100080, China; 2 School of Physics, University of Chinese Academy of Sciences, Beijing 100049, China; 3 Institute for Theoretical Physics, Chinese Academy of Sciences, Beijing 100190, China; Hong Kong University of Science and Technology, HONG KONG

## Abstract

Molecular dynamics (MD) simulation is an important tool for understanding bio-molecules in microscopic temporal/spatial scales. Besides the demand in improving simulation techniques to approach experimental scales, it becomes more and more crucial to develop robust methodology for precisely and objectively interpreting massive MD simulation data. In our previous work [J Phys Chem B 114, 10266 (2010)], the trajectory mapping (TM) method was presented to analyze simulation trajectories then to construct a kinetic transition network of metastable states. In this work, we further present a top-down implementation of TM to systematically detect complicate features of conformational space. We first look at longer MD trajectory pieces to get a coarse picture of transition network at larger time scale, and then we gradually cut the trajectory pieces in shorter for more details. A robust clustering algorithm is designed to more effectively identify the metastable states and transition events. We applied this TM method to detect the hierarchical structure in the conformational space of alanine-dodeca-peptide from microsecond to nanosecond time scales. The results show a downhill folding process of the peptide through multiple pathways. Even in this simple system, we found that single common-used order parameter is not sufficient either in distinguishing the metastable states or predicting the transition kinetics among these states.

## Introduction

Protein folding problem has been intensively studied for decades. Although in-depth understanding of proteins has been established by the pioneer works, see reference [1] for brief review, due to the tremendous complexity of these molecules, there is still a long way to get a clear and definitive description of conformational motions in proteins.

The current progress of experimental and simulation methods has made the protein structural ensemble accessible to researchers. In experiment, it is possible to directly observe the protein conformational dynamics by single molecular fluorescence method (SMF) [2]. Meanwhile, the full details of protein dynamics can be obtained by molecular dynamics (MD) simulations. The rapidly increasing computational power has enabled people to thoroughly study some small proteins with lots of parallel generated MD trajectories [3–7], or with a single long MD trajectory [8], up to milliseconds.

One intriguing point of proteins is that various folding intermediates generally exist [9, 10]. Besides, the unfolded ensemble of a protein also shows heterogeneity. In unfolded phase, proteins may have specific residue structures [11, 12] while statistically behaving like random coils [13]. The versatile metastable states of a polypeptide reflect the complexity of this molecule’s conformational space. A complete picture of protein dynamics can be established by explicitly identifying the metastable states and transitions between them. Such a picture may facilitate the study of some important protein molecules like intrinsic disorder proteins [14] and amyloid forming proteins [15].

So far, we have not provided a definition of a metastable state. A metastable state corresponds to a region in conformational space which is separated from other regions by high free energy barriers [16]. Consequently, a dynamics simulation trajectory entering into a metastable state will be trapped there in a characteristic timescale *τ*_*life*_, (*i.e.*, the lifetime of the state), which should be longer than the local equilibration timescale *τ*_*eq*_ in the state, (*i.e.*, the time that system loses its memory inside the state). Thus the kinetic transition between metastable states can be approximated as Markovian process [17]. Taking the states as nodes and transitions between them as edges, we can establish a transition network as a simplified picture about conformational motions of system [18–20].

To identify the states, the traditional way is to project simulation data to a low-dimensional space spanned by one or two manually selected order parameters, reconstruct the free energy contour map and then visually pick out the free energy minima (or basins) as states [21]. Some advanced techniques are also invented or applied to better select the order parameters [22–26]. However, it has been realized that the low-dimensional projection is usually not sufficient in complex systems, some metastable states may overlap each other after the projection, leading to artificial and distorted understanding in kinetics [27, 28]. In view of this, some methods have been designed to construct the transition network without the low-dimensional projection. Earlier attempts used clustering algorithms to directly group geometric similar conformations as metastable states [29–33]. However, metastable states should be defined on similarity of conformations in kinetics (or dynamics) rather than in geometry. In bio-molecules, structurally (geometrically) similar conformations may not be kinetically close to each other, and the structural difference of conformations inside a kinetic state may look not smaller than that of conformations in different states, (*i.e.*, the intra-state conformational fluctuation could be not smaller than the inter-state fluctuation).

Recently, a popular approach in classifying kinetic metastable states and transitions is the Markov state model (MSM) [34–42]. In the MSM, the sampled conformations are first classified into lots of small groups called microstates wherein the conformations are similar in geometry. As long as the partition of simulation samples is fine enough, the kinetics between microstates would be supposed as a discrete-time-discrete-state Markov process, and a transition rate matrix between microstates could be established by directly counting transition events along simulation trajectories. Then these microstates are further grouped into metastable states based on the standard spectral clustering method, and the transition network is constructed accordingly [34–42]. In the MSM, to ensure the correctness of the results, the number of microstates is often very large [43], but can not too large for getting sufficient number of transition events between them to estimate the transition rates.

In the previous works [19, 20], we proposed a trajectory mapping (TM) method to identify metastable states without a complete breakdown of simulation data. In the TM, we cluster simulation trajectory pieces rather than individual conformations, by mapping each trajectory piece as a high-dimensional vector with the average values of a set of (analytical) basis functions in the piece as components. The similar trajectory-mapped vectors are then grouped as metastable states, transition events in simulation trajectories are further identified. Recently, the idea describing conformational motions by analytical basis functions is also applied to improve and generalize the original MSM where a sample-based discrete functions are applied to describe the conformational motions. The benefits about the application of analytical basis functions are widely discussed [41, 42, 44–47]. For example, Nüske *et al*. [46] use the variational approach to relate the cross time-correlation matrix of analytical basis functions to a finite-dimensional approximation of dynamics propagator of systems, then the first eigenvectors of the correlation matrix provides slow dynamics modes. Another similar approach is called Time-Structure Based Independent Component Analysis (tICA) [44] which generalizes the usual principle component analysis (PCA) to relate the eigenvectors of the time correlation matrix to independent modes.

In this paper, we further improve our previous TM by presenting a hierarchical analysis strategy and a robust clustering algorithm to identify metastable states and transition network from the trajectory-mapped vectors in general. The state-searching process is now fully automated, and the complex transition network can be easily constructed accordingly in polypeptide. We also briefly discuss the relation between TM and the MSM-like methods.

## Materials and Methods

### Overview of trajectory mapping

Trajectory mapping (TM) is an analysis framework to identify metastable states from simulation data and to construct the transition network between the states. The TM maps molecular dynamics (MD) trajectories or trajectory pieces with approximately equal length *τ* to high-dimensional vectors,

$$\begin{array}{c} {\vec{v}}_{i}={\left(1,{\left\langle {\hat{A}}^{1}\left(q\right)\right\rangle }_{i},{\left\langle {\hat{A}}^{2}\left(q\right)\right\rangle }_{i},\ldots ,{\left\langle {\hat{A}}^{n}\left(q\right)\right\rangle }_{i}\right)}^{T}, \end{array}$$ (1)

where the components of the mapped vectors are the average values of conformational functions $\left\{{\hat{A}}^{\mu }(q)\right\}$ (named as basis functions) in the trajectory pieces. ${\langle {\hat{A}}^{\mu }(q)\rangle }_{i}=\frac{1}{\tau }\int_{0}^{\tau }{\hat{A}}^{\mu }(q_{i}(t)dt$. *q* denotes the conformational coordinates of the simulated system, such as the spatial positions of all atoms. *q*_*i*_(*t*) means the *i*th trajectory piece within the time interval *t* ∈ [0, *τ*]. Here the first basis function ${\hat{A}}^{0}(q)\equiv 1$ was explicitly written, all the other basis functions $\{{\hat{A}}^{\mu }(q)\},\mu =1,\cdots ,n$ are applied to describe (interested) conformational motions.

In the TM, the basis functions are orthonormalized each other under a reference distribution *P*_*ref*_(*q*),

$$\begin{array}{c} {\left\langle {\hat{A}}^{\mu }\left(q\right){\hat{A}}^{\nu }\left(q\right)\right\rangle }_{ref}=\delta^{\mu \nu }, \end{array}$$ (2)

Here ⟨⋯⟩_*ref*_ represents the average over *P*_*ref*_(*q*), which is estimated in the corresponding finite-size sample. We could choose all of the sampled conformations in these trajectory pieces, or a relevant part of these conformations as the reference sample. It is easy to linearly combine the preselected basis functions to form a set of orthonormalized basis functions satisfied Eq (2) by standard methods such as the Gram-Schmidt process, or PCA.

Aggregations of the trajectory-mapped vectors are found to correspond to metastable states in the previous work [20]. In simpler cases where only a few (*n*_*s*_) metastable states exist, we reduce the mapped vectors into a low (*n*_*d*_ = *n*_*s*_−1) dimensional space by PCA, then directly identify the aggregated clusters as metastable states. However, in peptide or protein systems, there are usually lots of metastable states in various (and not well separated) time scales, more systematical implementation of the TM and robust clustering algorithms are needed.

### Mathematical and physical meaning of TM

Before introducing details of the improved TM, we discuss the mathematical and physical meanings behind the TM, on such as basis functions, reference distribution, the PCA reduction, clustering of mapped vectors, and the identification of transition events.

#### Basis functions

Basis functions should be chosen to identify typical conformational motions of systems. Some physical quantities, such as, in protein, the torsion angles of backbone, distances of residue pairs, number of native contacts, root mean square deviation from some particular conformations, hydrogen bonded energy, solvated energy, etc., are good candidates of basis functions. In addition, since we usually focus on large-scale conformational motions, some fast degrees of freedom, such as hydrogen atoms, bond oscillation, etc., are usually excluded as basis functions. It is more efficient to select functions in coarse-grained conformational space as basis functions. More discussions about basis functions can be found in our previous works [19, 20, 48], or in some current approaches of MSM, such as tICA and the variational approach [44–47] where basis functions are similarly selected to expand the dynamics propagator.

#### Similarity of trajectory pieces

We define the overlapping integral of two probability density functions *P*_*i*_(*q*) and *P*_*j*_(*q*),

$$\begin{array}{c} \left\langle i|j\right\rangle =\int \frac{P_{i}\left(q\right)P_{j}\left(q\right)}{P_{ref}\left(q\right)}dq, \end{array}$$ (3)

where *P*_*ref*_(*q*) is a reference probability density function. Although the overlapping integral may be sensitive to *P*_*ref*_(*q*), some qualitative results, such as the zero value of the overlapping integral means no overlapping, is not dependent on the selection of *P*_*ref*_(*q*). We usually choose *P*_*ref*_(*q*) including both *P*_*i*_(*q*) and *P*_*j*_(*q*) to make the definition be reasonable. In practical application, the integral is usually estimated by finite-size samples of these probability distributions rather than their analytical formulas. Since

$$\begin{array}{c} \frac{P_{i}\left(q\right)}{P_{ref}\left(q\right)}=\sum_{\mu =0,\cdots }{\left\langle {\hat{A}}^{\mu }\left(q\right)\right\rangle }_{i}{\hat{A}}^{\mu }\left(q\right), \end{array}$$ (4)

the inner product of trajectory-mapped vectors, which defined as

$$\begin{array}{c} {\vec{v}}_{i}\cdot {\vec{v}}_{j}=\sum_{\mu =0,\cdots }{\left\langle {\hat{A}}^{\mu }\left(q\right)\right\rangle }_{i}{\left\langle {\hat{A}}^{\mu }\left(q\right)\right\rangle }_{j}, \end{array}$$ (5)

is a good estimate of the overlapping integral. Here we include the first trivial basis function ${\hat{A}}^{0}(q)\equiv 1$ and require $\left\{{\hat{A}}^{\mu }(q)\right\}$ satisfies Eq (2).

We further define the scaled inner product (SIP),

$$\begin{array}{c} \mathrm{SIP}={\hat{v}}_{i}\cdot {\hat{v}}_{j}=\cos\theta \left(i,j\right). \end{array}$$ (6)

Here $\hat{v}$ is the unit vector of $\vec{v}$. Therefore, while *i* and *j* correspond to two trajectory pieces which visit in the same metastable state and reach local equilibrium inside the state, their SIP is almost unity. Conversely, if the trajectory pieces *i* and *j* visit two complete different conformational regions without any overlapping, their SIP is almost zero. The value of SIP between zero and unity corresponds to the fact that the trajectories partially overlap in conformational space. In practice, although it is possible the SIP is slightly smaller than zero due to the finite sizes of samples and finite basis functions, it provides a good measure about similarity of trajectories. In the paper, we use the SIP (or it corresponding distance such as $d=\sqrt{2(1-\text{SIP})}$) rather than the usual Euclidean distance $d_{e}=\left|{\vec{v}}_{i}-{\vec{v}}_{j}\right|$ to measure the similarity of trajectory pieces. It is one of key points in the improvement of the TM.

#### Reduction of trajectories

There are closely relation between the TM and the variational approach [46] and the tICA [44]. The variance-covariance matrix element of $\left\{{\vec{v}}_{i}\right\}$ is

$$\begin{array}{ccc} {\bar{\Sigma }}_{\mu \nu } & = & \frac{1}{m}\sum_{i}{\left\langle {\hat{A}}^{\mu }\right\rangle }_{i}{\left\langle {\hat{A}}^{\nu }\right\rangle }_{i}\\ & = & \frac{1}{\tau }\int_{0}^{\tau }\;dt\left(1-\frac{t}{\tau }\right)\left[C_{\mu \nu }\left(t\right)+C_{\nu \mu }\left(t\right)\right]. \end{array}$$ (7)

Here $C_{\mu \nu }(t)=\frac{1}{\tau -t}\int_{0}^{\tau -t}dt_{1}\frac{1}{m}\sum_{i}{\hat{A}}^{\mu }(q_{i}(t_{1})){\hat{A}}^{\nu }(q_{i}(t_{1}+t))$ is nothing else but the time correlation in the variational approach [46], where the first (left or right) eigenvectors of the cross time-correlation matrix of basis functions correspond to slow dynamics modes (*i.e.*, the transitions between metastable states). The eigenvalues are expected to be single-exponential decay functions of time, while the basis functions are orthonormalized under the equilibrium distribution *P*_*eq*_(*q*). In the TM, we do not require to apply *P*_*eq*_(*q*) as the reference, and the variance-covariance matrix of trajectory-mapped vectors is a kind of average of the time correlation matrix. Although the principle components may not directly give slow modes, they well distinguish metastable states then provides the slow modes of system.

#### Clustering trajectories to states

As we already mentioned, a conformational region is a metastable state if the local equilibrium time inside the region, *τ*_*eq*_, is smaller than the life time of trajectory inside the region, *τ*_*life*_. We might measure the metastability of a state by the two times, such as $\kappa =\frac{\tau_{life}}{\tau_{eq}}$. In the TM, we map trajectory pieces with the length *τ* then cluster them as metastable states, thus some states can be found if they satisfied the condition,

$$\begin{array}{c} \tau_{eq}\leq \tau \leq \tau_{life}. \end{array}$$ (8)

On the one hand, if *τ* ≤ *τ*_*life*_, the *τ*−length trajectory pieces have significant possibility stay inside this state for identifying. Otherwise, trajectory pieces could only partially stay inside the state, thus no such a cluster corresponding to the state could be found. For these states, we can cut trajectories into shorter pieces (smaller *τ*) to make them be visible. On the other hand, *τ*_*eq*_ ≤ *τ* is a more basic condition in the TM, which ensures that all *τ*-length trajectories inside the state are mapped in the same cluster.

In realistic systems, there are usually lots metastable states with wide-distributed *τ*_*eq*_ and *τ*_*life*_. It is not easy to find all of them in a single *τ*. In this work, we first find large-size clusters at large *τ*, which obviously correspond to metastable states, then we cut the remaining trajectory pieces shorter and repeat to find large-size cluster as states, until most of data are identified or the remained trajectory pieces are too short.

#### Identify transition events

After finding metastable states, we can further translate simulation trajectories to state-indicator curves. These curves give the states that individual conformations (or a few successive conformations) located in. Concretely speaking, given *n*_*s*_ identified metastable states whose mapped vectors based on Eq (1) are denoted $\{{\vec{v}}_{\alpha }^{s}\},\alpha =1,\ldots ,n_{s}$, a simulation trajectory *i* can be transformed into *n*_*s*_ state-indicator curves, {*f*_*iα*_(*t*)},

$$\begin{array}{c} f_{i\alpha }\left(t\right)={\hat{v}}_{\alpha }^{s}\cdot {\hat{v}}_{i}^{\left[t-\Delta t,t+\Delta t\right]}. \end{array}$$ (9)

Here, ${\hat{v}}_{\alpha }^{s}$ is the unit vector of ${\vec{v}}_{\alpha }^{s}$, and ${\hat{v}}_{i}^{\left[t-\Delta t,t+\Delta t\right]}$ denotes the unit vector mapped from the conformations of the *i*th trajectory within the time interval [*t*−Δ*t*, *t*+Δ*t*]. If Δ*t* → 0, only the individual conformation *q*_*i*_(*t*) (the conformation of the *i*th trajectory at time *t*) is considered. Using finite Δ*t*, the statistical noise in the state-indicator curves could be depressed. Ideally, *f*_*iα*_(*t*) should be either zero or unity, *i.e.*, *f*_*iα*_(*t*) ≈ 1, if *q*_*i*_(*t*) ∈ *S*_*α*_, otherwise *f*_*iα*_(*t*) ≈ 0. Here *S*_*α*_ represents the state *α*. Therefore, the transition events between metastable states can be identified from the state-indicator curves.

### The systematical implementation of TM

#### The TM algorithm

We summarize algorithm of the TM as,

1. Choose a set of conformational functions and a reference sample, then form the othonormalized basis functions $\{{\hat{A}}^{\mu }\},\mu =1,\cdots ,m$.
2. Map trajectory pieces with length *τ* to vectors $\left\{{\vec{v}}_{i}\right\}$, and reduce the mapped vectors by PCA.
3. Group the mapped and reduced vectors by a clustering algorithm and identify larger cluster as metastable states.
4. Cut trajectory pieces which are not identified yet to shorter pieces, repeat the step 1 to 3 until the remained trajectory pieces are sufficient short.

Here, it is allowed to reset basis functions and the reference sample while varying time scale *τ* to better focus on the remaining simulation data.

#### The clustering algorithm

We briefly summarize the clustering algorithm as,

1. The trajectory-mapped vectors are grouped into clusters if their SIPs are larger than *r*_*l*_.
2. A cluster is identified as a metastable state if its size (number of vectors inside) is larger than $N_{ne}^{std}$.

Here we use the SIP defined in Eq (6) to measure the similarity of trajectory pieces. The SIP is almost within [0, 1] while sufficient basis functions are applied. It closes to unity if trajectories visit same conformational region, but zero while visiting completely different regions. Thus it is easy to set criterions to judge if two trajectories are similar. In this paper, *r*_*l*_ is set as 0.95, $N_{ne}^{std}=5$. The threshold of cluster size is used to exclude occasional concentrations of trajectory-mapped vectors. For example, we might generate two trajectory pieces which visit two metastable states occasionally with similar fractions in the two states, their conformational distributions are similar then the two pieces are mapped in one cluster. However, the probability to generate many trajectories with similar distributions but not in a single metastable state is small. The application of a larger threshold can depress the misjudgement while it might miss some metastable states. Since we will cut the non-identified trajectories into short pieces and repeat the clustering and state-identification process, the missed states will be found in the shorter time scales. Therefore, although the found states in each special *τ* may be dependent on the parameter $N_{ne}^{std}$, the final results of TM is not sensitive to that. In practical application, some additive judgements and tricks are also used to refine results. These details are listed in S1 Text of Supporting Information. We also illustrate the clustering algorithm in an imaginary models in Supporting Information as S1 Fig.

#### The hierarchical analysis strategy

Polypeptides are quite heterogeneous systems. There are many metastable states with various *τ*_*life*_ and *τ*_*eq*_, and there there could also be some sub-states inside states. The versatile stability and complex interrelation between metastable states reflect the hierarchical structure of a protein’s conformational space. In view of this, we designed a hierarchical analysis strategy and illustrate it with an imaginary example in Supporting Information, see S2 Fig.

### Simulation and analysis details

In the paper, we apply the TM in alanine-dodeca-peptide [Ala_12_], a polypeptide composed of 12 alanine residues. The simulation is performed with TINKER4.2 package using OPLSUA force field and GB/SA implicit solvent model [49]. Charged termini in Ala_12_ are used, which leads to versatile metastable structures [35]. The conformations are recorded every 0.5 ps. In the previous work [20], we studied this molecule with 1000 20-ns length simulation trajectories. We found that most of the identified metastable states correspond to *β*-hairpin/coil conformations, and *α*-helix conformation is less stable then *β*-hairpin/coil conformations using current force field, which is consistent with previous experimental and theoretical results [50, 51]. Owing to the limited simulation length of each trajectory and the tentative clustering algorithm, previously we did not globally analyze the system, but only focused on some local structures instead. In this work, five 4μs-length simulation trajectories were generated, one of the five trajectories is spawned from *α*-helix conformation, and all the others were initiated from *β*-hairpin/coil conformations to reflect the relative importance of these conformations.

We select the functions of backbone *ϕ* and *ψ* angles as basis functions. Here *ϕ* is defined as the backbone dihedral angle around the bond connecting C_*α*_ and N atoms, *ψ* is defined as the backbone dihedral angle around the bond connecting C_*α*_ and carbonyl carbon atoms. There are 22 *ϕ* or *ψ* angles in Ala_12_. These angles fully account for the backbone flexibility of this molecule. They are transformed into basis functions using the following two-dimensional trigonometrical functions.

$$\begin{array}{ccc} & \sin[(m+n)x],\cos[(m+n)x], & \\ & \sin[(m+n)y],\cos[(m+n)y], & \\ & m+n>0 & \\ & \sin(mx)\sin(ny),\sin(mx)\cos(ny), & \\ & \cos(mx)\sin(ny),\cos(mx)\cos(ny), & \\ & m\geq 1,n\geq 1 & . \end{array}$$ (10)

Here *x* and *y* are two angles measured in radius. *m* and *n* are non-negative integers. We define the summation of *m* and *n* in Eq (10) as the order of these functions, and use the one-to-two order functions in analysis. Only the correlation between sequentially neighboring dihedral angles are modeled by the basis functions. Therefore, 172 basis functions are finally included in analysis. 88 of them are functions of single dihedral angles and the remaining 84 ones are functions of neighboring dihedral angles. For peptide system, this set of basis functions is already enough for a reasonable estimation in the TM [19, 20]. It should be noted that it is possible to select functions of other degrees of freedom (such as inter-atomic distances) or of carefully chosen collective variables in analysis, similar results could be obtained. The backbone dihedral angle is a simple and natural choice for describing the global conformational motions of peptides [52].

We performed the hierarchical analysis at three timescales. The trajectories are first truncated to 100 200ns-length trajectory pieces. After clustering, the trajectory pieces that are not allocated to any metastable state are truncated to 20ns-length, the shorter pieces that are not overlapping to existing metastable states are picked out for next round of clustering. The non-allocated ones in this round of clustering are truncated to 2ns-length. Then, the 2ns-length trajectory pieces that are not overlapping to existing metastable states are kept for the final round of clustering.

## Results

### The metastable states of Ala_12_

We first examined the convergence of the five 4*μs*-length simulation trajectories. We calculated the similarity between the conformational distributions of these long trajectories, *i. e.* the scaled inner product (SIP) defined in Eq (6). The results are shown in Supporting Information as S3 Fig. Although every simulation trajectory partially overlaps with some others, there do not exist two simulation trajectories very similar to each other such that their SIP is close to one. Therefore, the SIP measure clearly shows that none of the long simulation trajectories has reached the global equilibrium. We need to combine the information in these trajectories to get a synthesized picture of the system by the TM.

#### Metastable states

Through hierarchical analysis at three levels, 28 states were automatically found by the TM. We first found 2 states at 200ns timescale, then 11 states at 20ns timescale from the remained parts of trajectories, finally, 15 states at 2ns timescale. These states are further refined to ensure that the SIP values between different states are almost zero (smaller than 0.01 in the current analyses). The identified metastable states are orthogonal to each other, *i.e.* different states are not overlapping in conformational space, indicates our basis functions is sufficient to completely distinguish these states. The SIPs among states are shown in Supporting Information as S4 Fig.

#### Transitions between states

**Fig 1:**
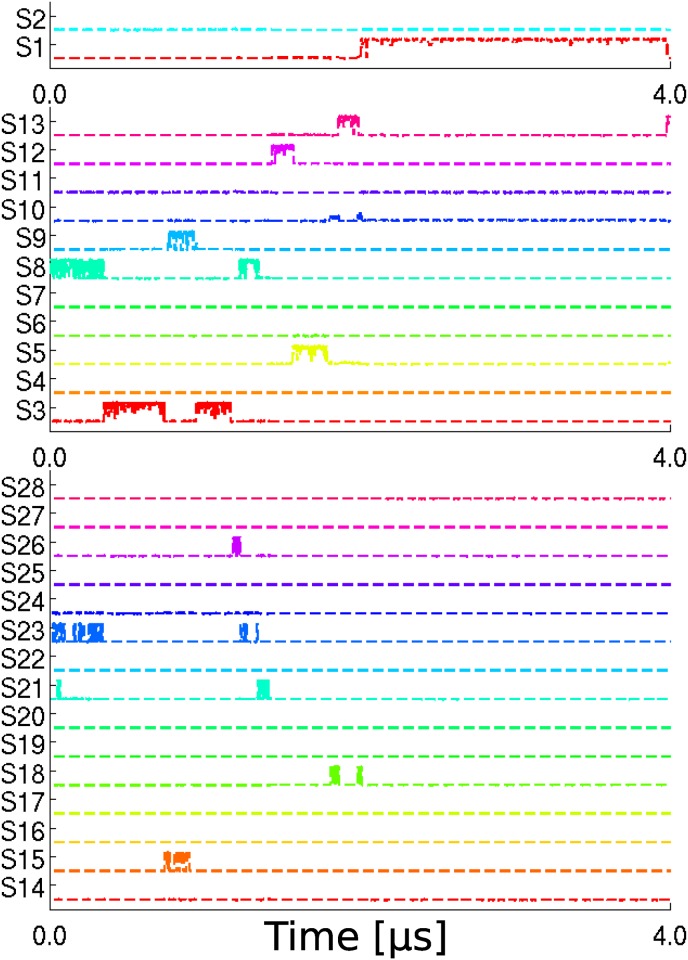
The 28 state-indicator curves of the 3rd 4μs-length trajectory. The curves are divided into three groups according to the identified timescale of corresponding states. The states are numbered according to the sequence of finding.

**Fig 2:**
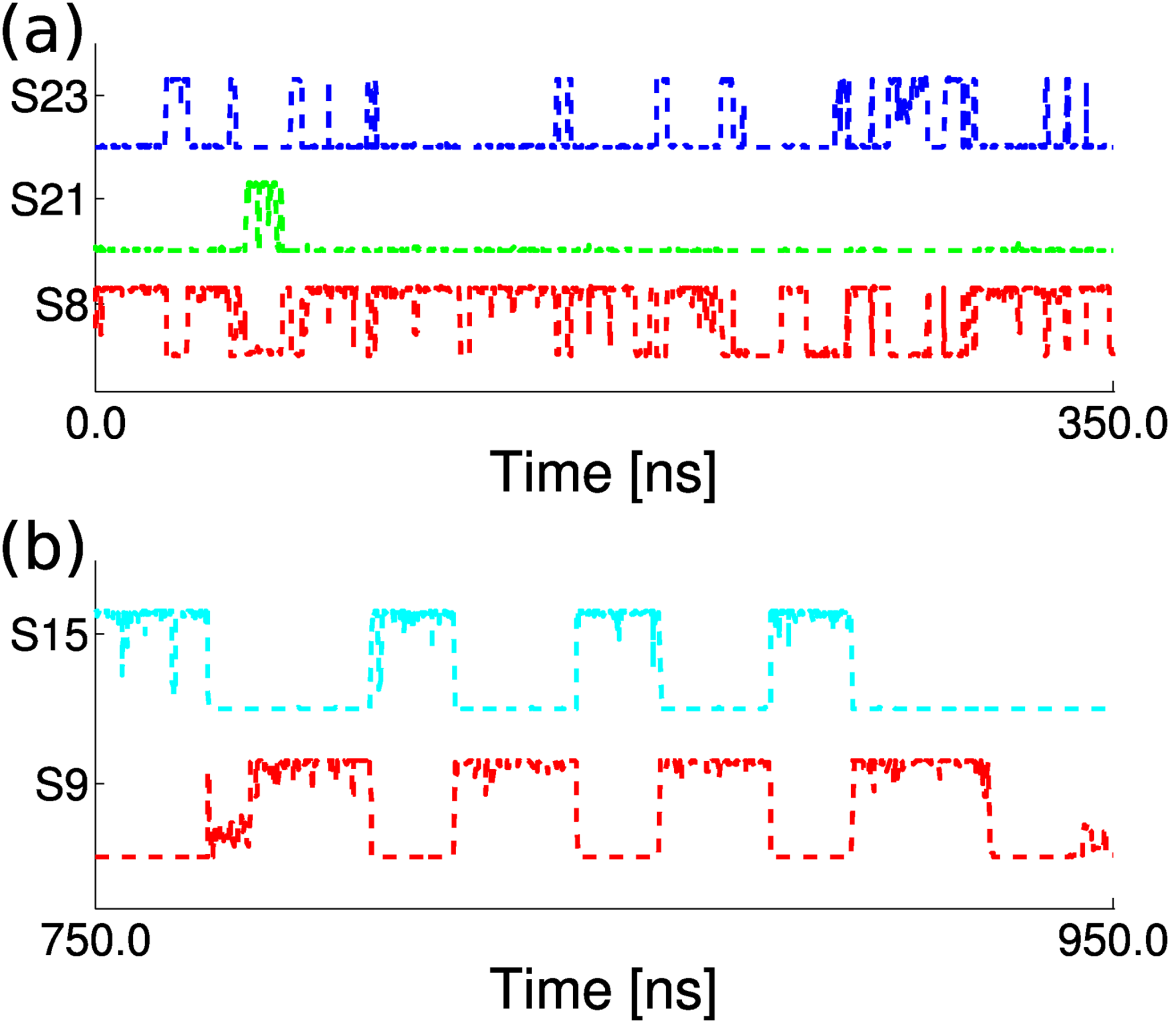
The detailed view for some of the state-indicator curves in the trajectory.

We can get the state-indicator curves by projecting the simulation trajectories to the 28 states. A set of representative state-indicator curves are plotted in Fig 1. The others are shown in Supporting Information as S5, S6, S7 and S8 Figs. There are totally 28 curves, each addresses the occupation timing and fraction of the third 4μs trajectory in a metastable state. As we mentioned, if the simulation trajectory stays in certain state around certain time, the state-indicator curve of this state should take a value close to 1.0 at this moment. Thus, it could be deciphered from Fig 1 that the 4μs trajectory started off from state *S*_8_, after traveling around other 10 states, it finally entered state *S*_2_ and stayed there for the last 2μs, which indicates the lifetime of state *S*_2_ is at least in μs-scale. We also provide the enlarged view of the parts of trajectory from 0ns to 350ns and from 750ns to 950ns in Fig 2. During the first period, the trajectory quickly traveled among state *S*_8_, *S*_21_ and *S*_23_. During the second period, the trajectory jumped between state *S*_9_ and *S*_15_. It can be seen that the state indicator curves usually show step-like behavior, jumping between value 0 and 1, which shows that the transitions between states are quite fast compared to *τ*_*eq*_ and *τ*_*life*_. There also exist some regions in which the state-indicator curve takes value between zero and one, for example, see the curve of *S*_9_ in Fig 2(b). In that case, the trajectory may enter a conformational region which does not have a good metastability then is called as a diffusive-like region. Sometimes all the state-indicator curves take almost zero value shortly, which indicates that there are local unidentified regions which could either be metastable or diffusive. The above-mentioned abnormality of state-indicator curves is understandable considering the complexity of bio-molecules’ conformational space.

We list the number of trajectory pieces used for defining the metastable states in Supporting Information, see S1 Table. For each state, we also list the average SIP value between the defining trajectory pieces and the representative vector of the state. An average SIP value close to 1.0 indicates that the trajectory pieces in a state resemble each other quite well. To calculate the average SIPs, *P*_*ref*_(*q*) is selected as the equal weight linear combination of the identified metastable states. In S2 Table of Supporting Information, we show the proportion of conformations which are identified as metastable states in the five 4μs simulation trajectories. More than 90 percent of the simulation data is found to stay in the identified metastable states, which suggests the remarkable metastability of Ala_12_. Among the five simulation trajectories, the fifth trajectory is least accounted by metastable states, this is because the final part of this trajectory entered into a large region with a few metastable states and some other small regions with prominent diffusive behavior inside (*i.e.*, *κ* = *τ*_*life*_/*τ*_*eq*_ is not obviously larger than unity).

#### Metastability of states

**Fig 3:**
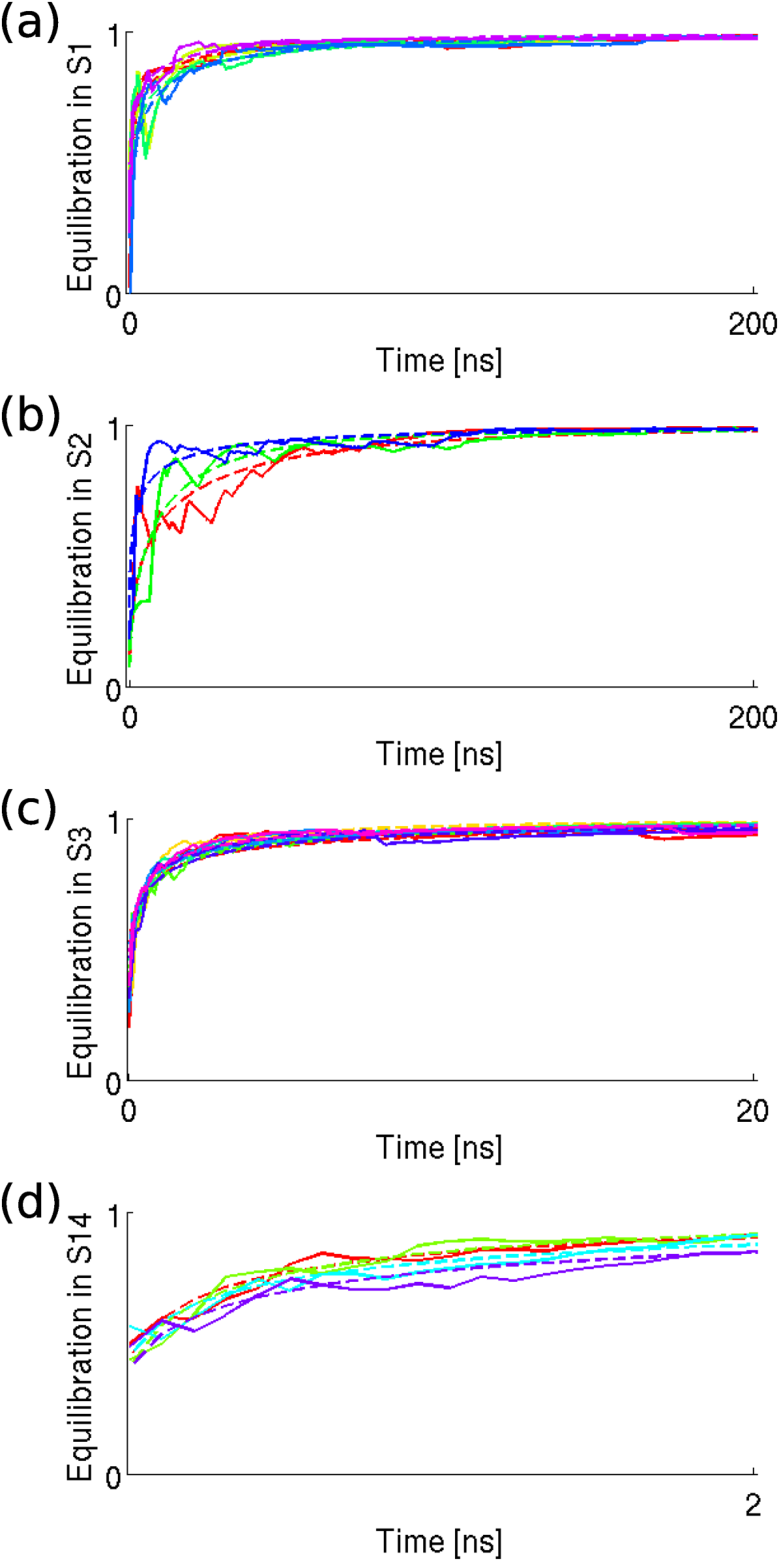
The equilibration process in metastable states. In each panel, different color represents different trajectory pieces used to estimate the equilibration process. The solid lines are calculated with simulation data, the dotted lines are the fitted stretched exponential curves.

We also tested whether the 28 metastable states satisfy the assumption *τ*_*eq*_ < *τ*_*life*_. To estimate the *τ*_*life*_ of a metastable state, we picked out all the trajectory pieces that continuously stay in this state, and took their average length as an estimation. To estimate the *τ*_*eq*_ of a metastable state, we calculated the relaxation behavior of the *τ*-length trajectory pieces defining the state. Concretely speaking, for each trajectory piece *i* defining the state *S*_*α*_, the SIP between the conformations in *S*_*α*_ and the conformations of the beginning *u*-length part of trajectory *i*, are calculated for *u* ∈ [0, *τ*]. The SIP should be small when *u* is close to zero. Meanwhile, as increasing *u* to approach to *τ*_*eq*_, it approaches to 1 within statistical error. Such this kind of SIP curve illustrates the relaxation of a trajectory to the local equilibrium inside a state. We plot some SIP curves for the states *S*_1_, *S*_2_, *S*_3_ and *S*_14_ in Fig 3. The SIP curves are fitted with the stretched exponential model [53].

$$\begin{array}{c} {\hat{v}}_{\alpha }^{s}\cdot {\hat{v}}_{i}^{\left[0,u\right]}=c\left\{1-\exp\left[-{\left(au\right)}^{b}\right]\right\}. \end{array}$$ (11)

**Fig 4:**
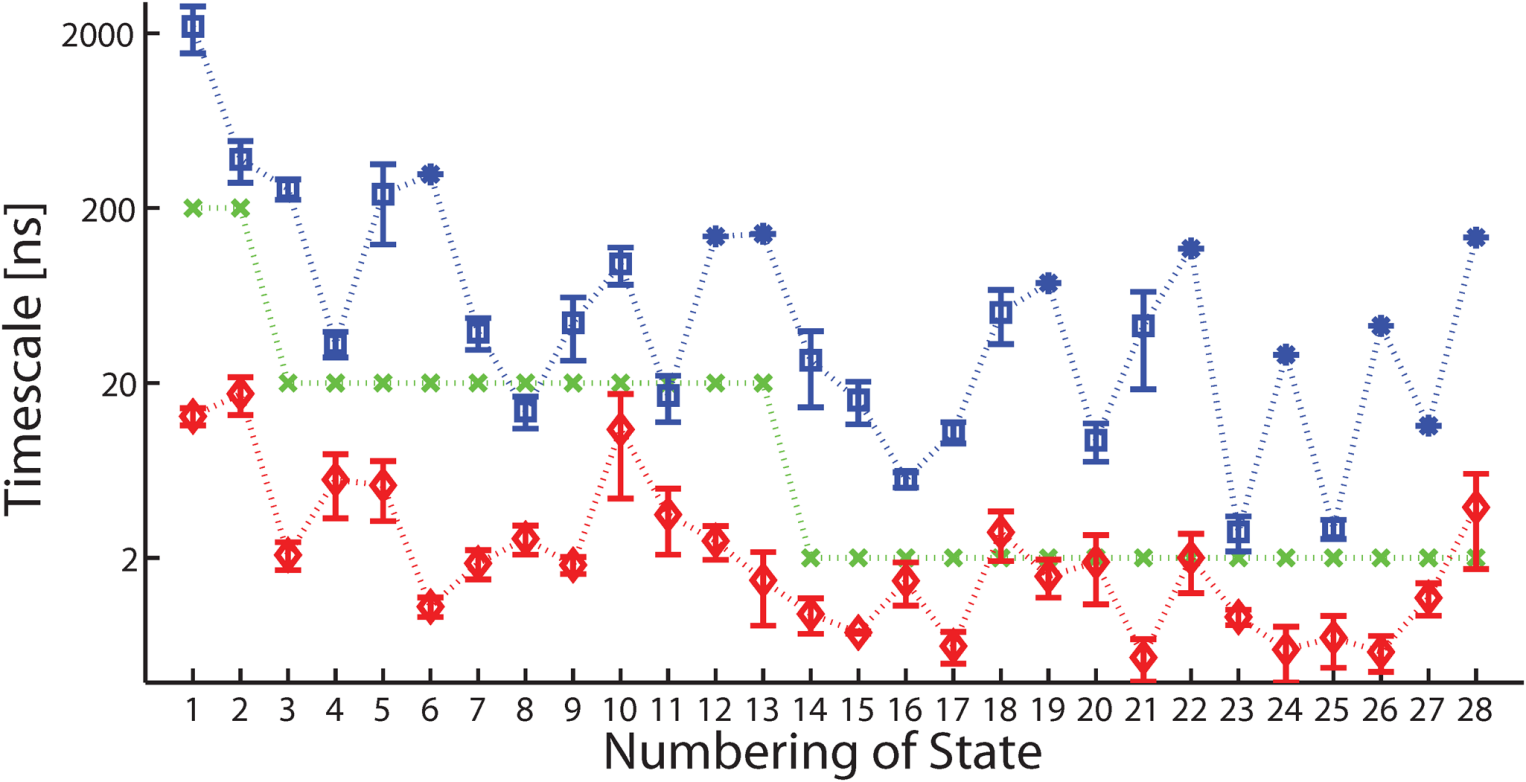
Comparison between *τ*_*life*_, *τ* and *τ*_*eq*_. The blue symbols (squares for the ones with error bar, stars for the ones without error bar) denote the estimated *τ*_*life*_ of the metastable states. The green crosses denote the identified timescale of the states. The red diamonds denote the estimated *τ*_*eq*_. The error bars are estimated where possible. The dotted lines are just for aiding the inspection.

For each trajectory piece, an estimation of *τ*_*eq*_ can be obtained by

$$\begin{array}{c} \tau_{eq}^{est}=\frac{1}{ab}\Gamma (\frac{1}{b}), \end{array}$$ (12)

where Γ(*x*) is the Gamma function. Averaging the $\tau_{eq}^{est}$ values of the trajectory pieces defining a state leads to the estimation of *τ*_*eq*_ of that state. The final results of *τ*_*eq*_ and *τ*_*life*_ are shown in Fig 4. The relation *τ*_*eq*_ < *τ*_*life*_ is indeed satisfied within statistical error.

#### Test of the inner product estimate

We further demonstrate the validity of Eq (5). The evolution of probability distribution are often thought to follow the multiple-dimensional Fokker-Planck Equation, $\frac{\partial P(q,t)}{\partial t}=\text{L}P(q,t)$, where **L** is the Fokker-Planck operator. We have

$$\begin{array}{c} P\left(q,t\right)=\phi_{0}\left(q\right)\sum_{n=0,1,\ldots }C_{n}\phi_{n}\left(q\right)\exp\left(-\lambda_{n}t\right), \end{array}$$ (13)

where {*ϕ*_*n*_(*q*)} is orthonormalized, *i.e.*, ∫*ϕ*_*n*_(*q*)*ϕ*_*m*_(*q*)*dq* = *δ*_*n*, *m*_, and ∣*ϕ*_0_(*q*)∣^2^ = *P*_*eq*_(*q*), the equilibrium distribution. The non-negative {*λ*_*n*_} is sorted from small to large, *λ*_0_ = 0. The expansion coefficient {*C*_*n*_} is determined by the initial distribution *P*(*q*, *t* = 0). We define the average distribution

$$\begin{array}{c} P_{avr}\left(q,t\right)=\frac{1}{t}\int_{0}^{t}P\left(q,t^{\prime }\right)dt^{\prime }. \end{array}$$ (14)

If choosing *P*_*ref*_(*q*) = *P*_*eq*_(*q*), it is straight forward to show the overlapping integral defined in Eq (3)

$$\begin{array}{c} \left\langle P_{avr}\left(t\right)|P_{avr}\left(t\right)\right\rangle =1+\frac{1}{t^{2}}\sum_{n>0}{\left(\frac{C_{n}}{\lambda_{n}}\right)}^{2}{\left[e^{-\lambda_{n}t}-1\right]}^{2}. \end{array}$$ (15)

As *t* increases, the exponential terms in Eq (15) decay to zero quickly, and ⟨*P*_*avr*_(*q*, *t*)∣*P*_*avr*_(*q*, *t*)⟩−1 will be proportional to 1/*t* ^2^.

**Fig 5:**
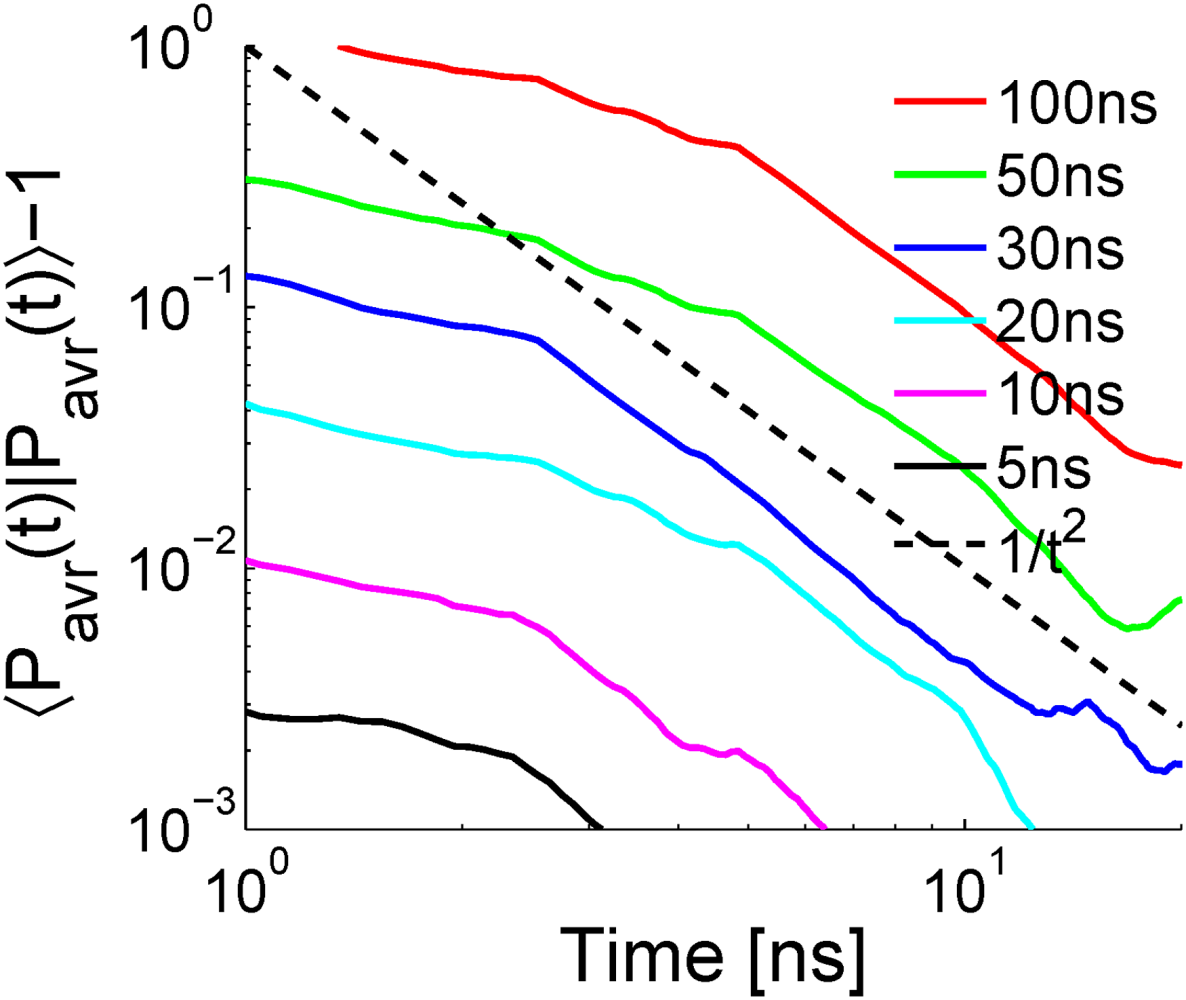
The scaling behavior of $\langle P_{avr}\left(\vec{q},t\right)|P_{avr}\left(\vec{q},t\right)\rangle -1$ versus *t*.

Due to the lack of global equilibrium sample, we focus on the local equilibrium inside the state *S*_1_, and use the local equilibrium sample as the reference. Since Eq (15) is related to the time relaxation of *P*_*avr*_(*q*, *t*), we truncate the trajectory pieces defining *S*_1_ to even shorter pieces and estimate the relaxation of the ensemble of the short trajectories. Short trajectories of lengths 100ns, 50ns, 30ns, 20ns, 10ns and 5ns are analyzed. In these cases, we have 52, 104, 156, 260, 520 and 1040 pieces in the trajectory ensemble respectively. The shorter the truncated trajectories, the initial distribution *P*(*q*,0) of the ensemble of trajectories is more similar to the local equilibrium distribution of *S*_1_. The relaxation behavior of ⟨*P*_*avr*_(*q*, *t*)∣*P*_*avr*_(*q*, *t*)⟩−1 estimated by Eq (5) is plotted in Fig 5. Apparently, all the curves shown in Fig 5 become proportional to 1/*t* ^2^, consistent with the theoretical result. Besides, the timescale at which the crossover to 1/*t* ^2^ behavior happens is also consistent with the estimated *τ*_*eq*_ of *S*_1_, see Fig 4. Therefore, the sample estimation of the inner product between two conformational functions, see Eq (5), is promising.

### The transition network and polypeptide folding

**Fig 6:**
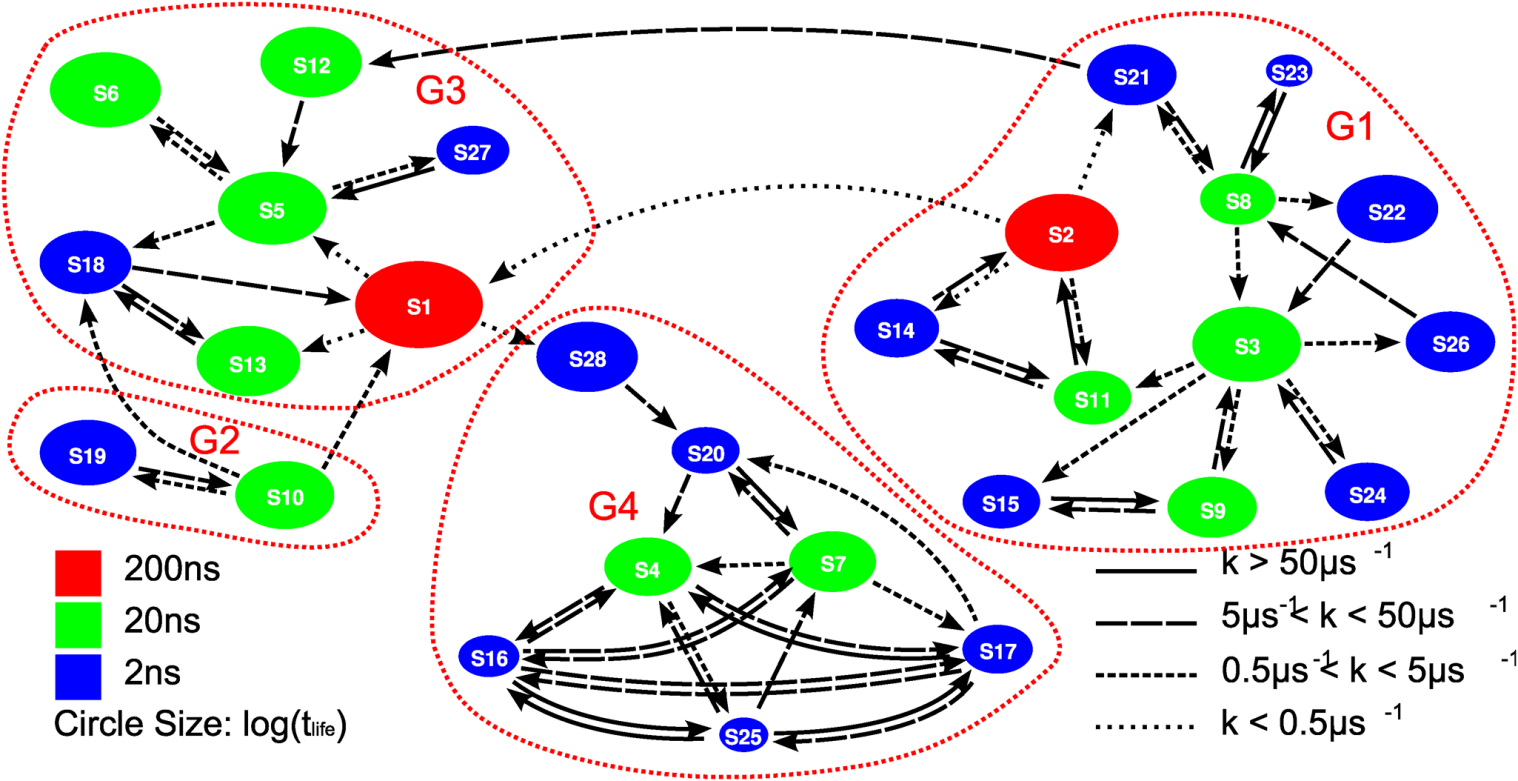
The transition network. Each node represents a metastable state. The states are colored according to their identified timescales, and their sizes are determined by the estimated *τ*_*life*_. The transitions between states are plotted with different line styles according to the estimated transition rates. The classification of the 28 states is also shown.

Based on the identified metastable states and the state-indicator curves, we derived out a transition network and plotted it in Fig 6. The metastable states are shown with circles of various size and color, they are connected to each other according to their transition relation. It should be noted that the transition relation illustrated here is inferred from the simulation data. If the transition between two states only happens in one direction in our finite-time simulation, the two states will only be connected by single-directional arrow. Thus the transition network may lack detailed-balance property due to finite sampling. Still, to get a qualitative picture, we estimated the rates of the observed transitions as follows. For each state *S*_*i*_, we estimated its lifetime $\tau_{life}^{i}$. Suppose the jump from *S*_*i*_ to another state *S*_*j*_ happened for *N*_*ij*_ times, the kinetic transition rate in this direction could be estimated by

$$\begin{array}{c} k_{ij}=\frac{N_{ij}}{\tau_{life}^{i}\sum_{j}N_{ij}}. \end{array}$$ (16)

The different line styles for the transition arrows in Fig 6 illustrate the magnitude of non-zero transition rates.

#### Simplified picture in *μs* scale

According to the transition relation, we roughly partition the 28 states into 4 groups, *G*_1_, *G*_2_, *G*_3_ and *G*_4_. The composition of the groups can also be found in Fig 6. In simulation, all the transitions between the states in different groups are found to be single-directional. Concretely speaking, we can only find the transitions from the states in *G*_1_ to the states in *G*_3_, from the states in *G*_2_ to the states in *G*_3_ and from the states in *G*_3_ to the states in *G*_4_, while all the reverse transitions didn’t show up. However, the states in the same group are kinetically closely related to each other. Except for *S*_12_ in *G*_3_ and *S*_28_ in *G*_4_, we can find transition routes of reverse directions between any two states in the same group. Meanwhile, *S*_12_ and *S*_28_ are actually small intermediate states for the transitions from *G*_1_ to *G*_3_ and from *G*_3_ to *G*_4_, respectively. Thus, the whole picture of the 28 states looks quite like the downhill folding of protein. The states in *G*_1_ and *G*_2_ represent two different kinds of denatured states, with a few μs occupying time. The states in *G*_3_ represent the intermediate states of folding. *G*_4_ seems the end of simulation dynamics. System enters the region and stays there more than 2.8μs without leaving. Although it is not clear if *G*_4_ is still only an intermediate region in much longer simulation, in the paper, we name the states in *G*_4_ might constitute the folded-state-like ensemble, partially because the conformational structure looks like the folded one.

#### The inner structure of two long lifetime states

The sub-states of the 200ns-order metastable states *S*_1_ and *S*_2_ in *Ala*_12_ can be found at nanosecond or sub-nanosecond scales. With *τ* = 2ns, we found four sub-states for both *S*_1_ and *S*_2_. The sample state-indicator curves of the sub-states of *S*_1_ and *S*_2_ are shown in Supporting Information, S9 and S10 Figs, respectively. (In the pictures, we use *S*_*a*(*b*)_ to denote the *b*th sub-state of state *S*_*a*_.) Compared with S6 Fig, the state-indicator curves of the sub-states indeed reflect the detailed inner-state dynamics of *S*_1_ and *S*_2_. Meanwhile, the state-indicator curves of the sub-states of *S*_1_ show much more prominent roughness than the ones of *S*_2_, which reflects the more diffusive nature of *S*_1_. The fast and sharp transition between the sub-states of *S*_2_ is consistent with our previous results [20]. The representative structures of the sub-states and their inner-relation are shown in S11 and S12 Figs of Supporting Information.

#### The persuit of reaction coordinate

**Fig 7:**
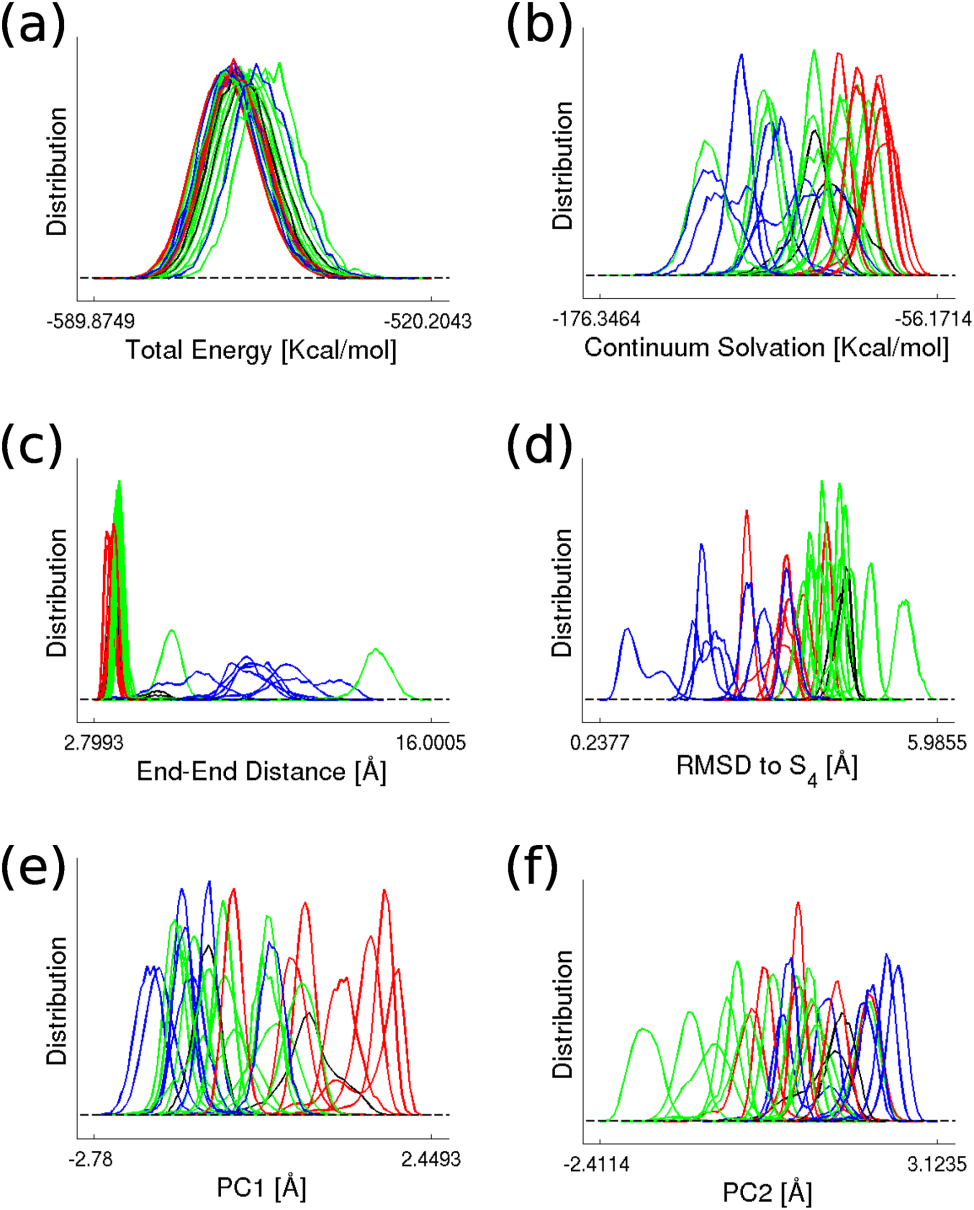
The distributions of the 28 states along various collective variables. The selected collective variable include total energy of the system (a), the solvation energy (b), the distance between the two ends of the peptide (c), the RMSD relative to a representative conformation in *S*_4_ (d), the first (e) and the second (f) principle component of dihedral angle principle component analysis [22].

Since we have made an analogy of the 28-state transition network to the downhill folding process, it is natural to ask whether there exists certain collective variable that can be used as the reaction coordinate of the system. In Fig 7, we plotted the probability distributions of the 28 metastable states along six collective variables. It can be seen that, while all the states have similar total energy distributions [see Fig 7(a)], their solvation energy distributions show conspicuous heterogeneity [see Fig 7(b)]. The folded-like states (states in *G*_4_) have relatively low solvation energy. On the contrary, the intermediate states (states in *G*_3_) have relatively high solvation energy. The unfolded states (states in *G*_1_ and *G*_2_, or might be denoted as partially folded states more exactly) have their solvation energy distributed in between. Therefore, in the “folding” process, the unfolded states are first transformed into more compact form such that the contact between the backbone polar residues and water is reduced. After that, the polar residues are released to solvent again, and the folded-like ensemble is stabilized by solvation energy. Although the solvation energy provides a qualitative standard to differentiate the states of different identities, it is not qualified to be a reaction coordinate. For one thing, the states in different groups have overlapping distributions of solvation energy. For another, the transition from unfolded states to folded states is not monotone along the solvation energy axis.

We also tested two commonly used reaction coordinates, the end-end distance between the two ends of Ala_12_ and the root mean square deviation (RMSD) to *S*_4_, where *S*_4_ is one of the states in the folded-like ensemble *G*_4_. As can be seen from Fig 7(c), the folded states have prominently larger end-end distance than most of the intermediate and unfolded states. Their distributions along the end-end distance axis also show much more variety than the others. On the contrary, the overlapping end-end distance distributions for most of the intermediate and unfolded states suggest that the two terminals of the molecule are closely restrained together in these states. The charged terminal adopted in current simulation help to stabilize such a close end-end contact. Although the end-end distance provides a clear separation of the folded state ensemble and the other states, it still can not provide the correct picture of the reaction process. Besides, the separation is probably owing to the nature of this system and can not be generalized. As shown in Fig 7(d), the RMSD to *S*_4_ seems more promising. The states in different groups are partially separated along the RMSD axis. Besides, the folding process happens with decreasing RMSD value. However, there still exist overlapping states from different groups. Thus, projecting the simulation samples to the RMSD axis may also lead to mis-interpreted kinetics.

Finally, we tested the principle components from dihedral angle PCA [22]. The distributions of the 28 states along the first and the second principle components are shown in Fig 7(e) and 7(f) respectively. Due to the strong overlap between states, the principle components can not help to clearly dissect the system into folded, intermediate and unfolded conformational ensembles.

In summary, we found that even for the simple system of Ala_12_, the state structure in conformational space is very complicated. It is very hard to select a single reaction coordinate to precisely reflect the complexity of the system. Multiple reaction coordinates are usually necessary. In the simple system, we may use two or three well-chosen reaction coordinates to distinguish all the metastable states, or one reaction coordinate may be sufficient in describing the transitions among a part of the metastable states, but generally, the network model shows its superiority for describing the complexity of bio-molecular systems.

#### The folded-like ensemble

**Fig 8:**
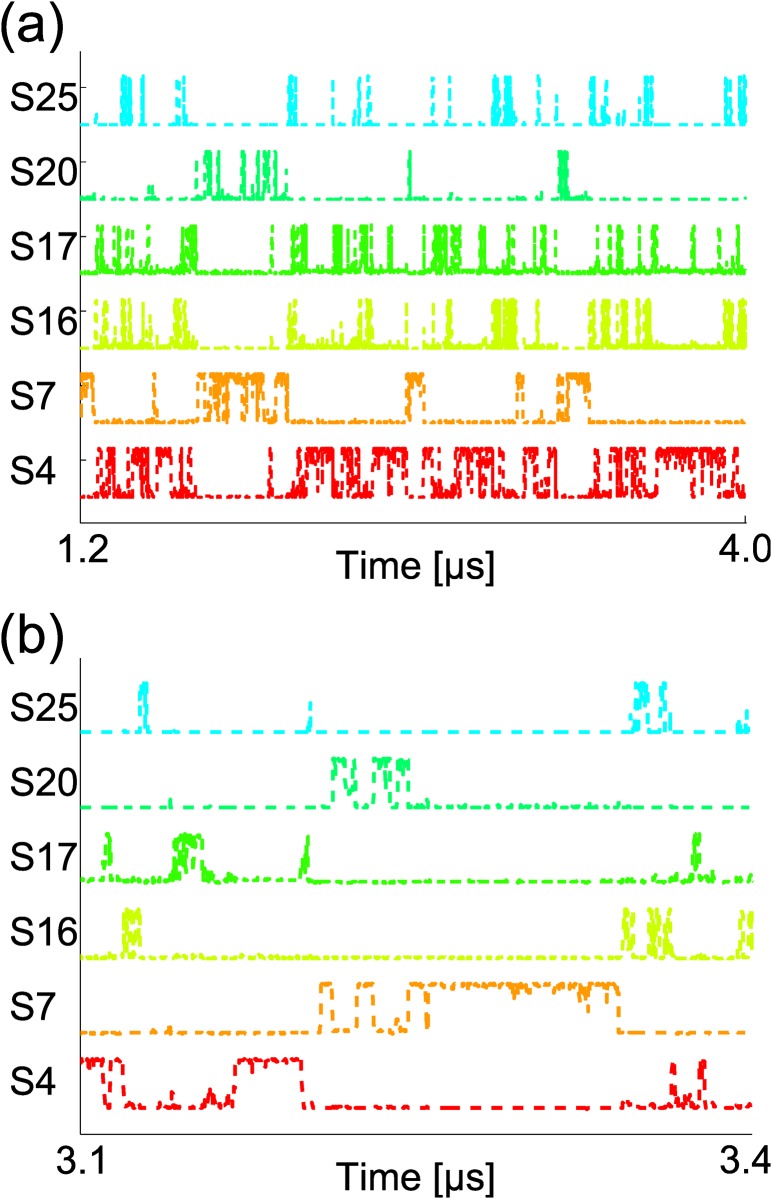
The state-indicator curves of *S*_4_, *S*_7_, *S*_16_, *S*_17_, *S*_20_ and *S*_25_ along the 5th trajectory. (a) shows the last 2.8 μ s. (b) provides an enlarged view from 3.1 μ s to 3.4 μ s.

**Fig 9:**
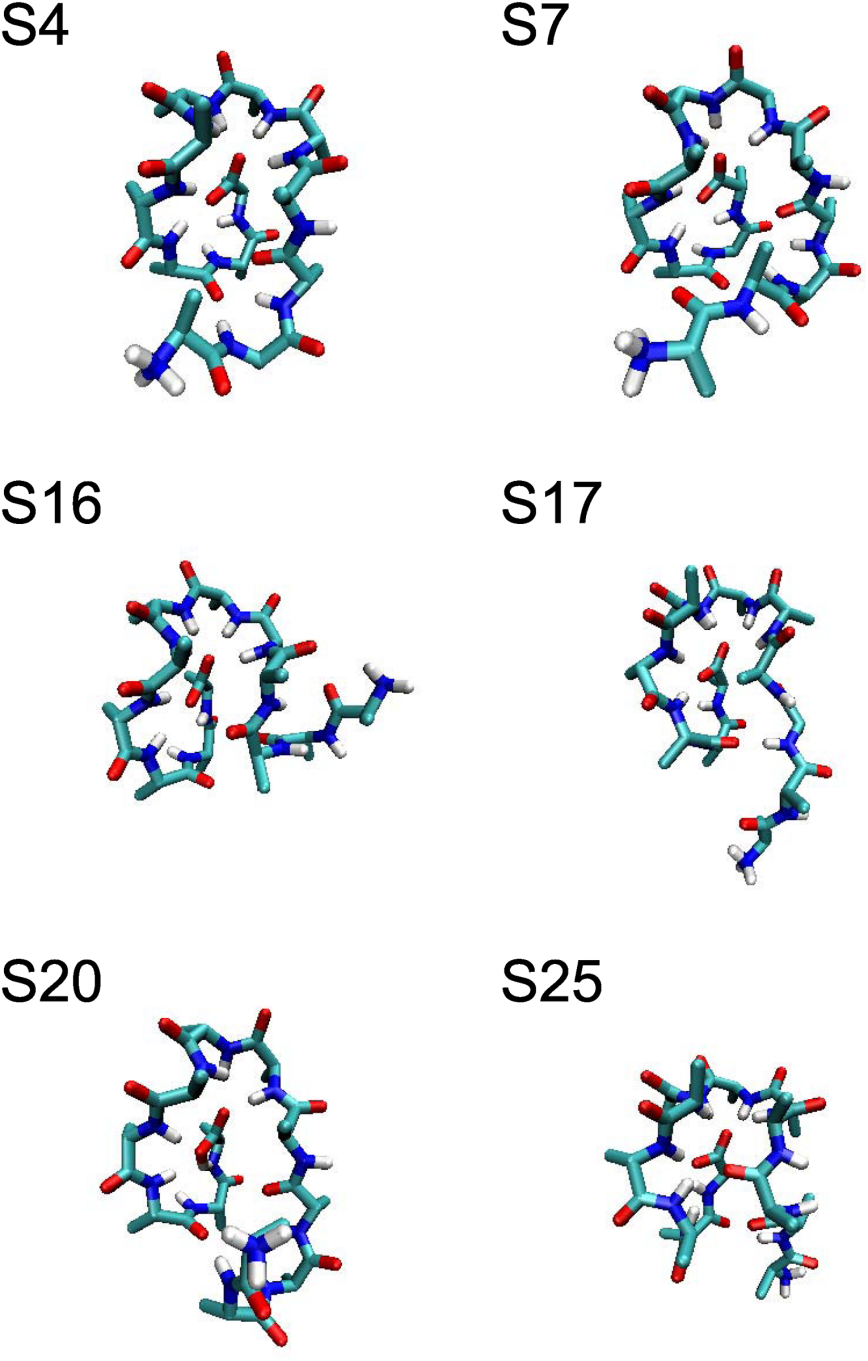
The representative structures of *S*_4_, *S*_7_, *S*_16_, *S*_17_, *S*_20_ and *S*_25_.

Now we focus on the folded-state ensemble, *i.e.* the states in *G*_4_ group. All the seven states in *G*_4_ lie in the end portion of the fifth 4μs trajectory. The trajectory first entered *S*_28_. After a short stay, it moved to a free energy basin containing *S*_4_, *S*_7_, *S*_16_, *S*_17_, *S*_20_ and *S*_25_, and jumped fast between these six states until the end of the simulation. The inter-state transitions between the six states are shown in Fig 8(a). This period lasts for 2.8 μ s. We found there is about 20 percent conformations of the 2.8 μ s trajectory unaccountable by the six metastable states, where none of the state-indicator curves of *S*_4_, *S*_7_, *S*_16_, *S*_17_, *S*_20_ and *S*_25_ is considerably larger than zero [see Fig 8(b)]. Of course, it is possible that we failed to find all the metastable states in this region. However we have tried to use different length *τ* of trajectory pieces to look for more detailed state structures, and there is no qualitatively change of the results. So it seems that the conformational region shows obvious diffusive behavior out of the six well-defined states. The representative structures of the six states also support the diffusive behavior inferred from the state-indicator curves. As shown in Fig 9, the six representative structures from these states have similar C-terminal structures and versatile N-terminal structures. In all the states, the C-terminal of Ala_12_ form stable hydrogen bonds with the amine bases in the middle of the chain. Meanwhile, the N-terminal chain is not confined by strong intra-molecular interactions. Such a flexible N-terminal leads to the diffusive-like property. Considering the fast inter-state transition between *S*_4_, *S*_7_, *S*_16_, *S*_17_, *S*_20_ and *S*_25_, it seems that these states as well as the outside diffusive regions connecting them constitute a large metastable state. Actually, the SIP between the first half and the second half of the 2.8 μ s-length trajectory has reached 0.88, which suggests the similarity between the two halves and consequently the local equilibration in this part of simulation trajectory. If the above guess is true, the *τ*_*eq*_ of the large metastable state should be several micron-second long, which is three orders of magnitude larger than the *τ*_*eq*_ of its sub-states. The separation of the equilibration timescales between a state and its sub-states generally exists. We also tried to find the sub-states of *S*_1_ and *S*_2_ with the TM method. The sub-states can only be found at nano-second or sub-nano-second scales (see the supplementary material). Actually, only when there is timescale separation, the *τ*_*life*_s of the sub-states could be prominently smaller than the *τ*_*eq*_ of the host state, which ensures the consistency of the identified metastable states, see Eq (8).

#### The characterization of conformational dynamics

For the transition network established by the TM, the local equilibrium sample of the states are obtained. Therefore, we can calculate the average value of any physical quantity within the states. This information could help to characterize the conformational dynamics of inter-state transitions. We considered the 22 flexible backbone dihedral angles used to define the basis functions in the TM. For each state, we calculated the averaged sine and cosine functions of these dihedral angles, and aligned the 44 values sequentially to form a vector. This vector characterizes the conformations of corresponding state. When comparing the difference between vectors in different states, some clues of transition dynamics could be obtained.

**Fig 10:**
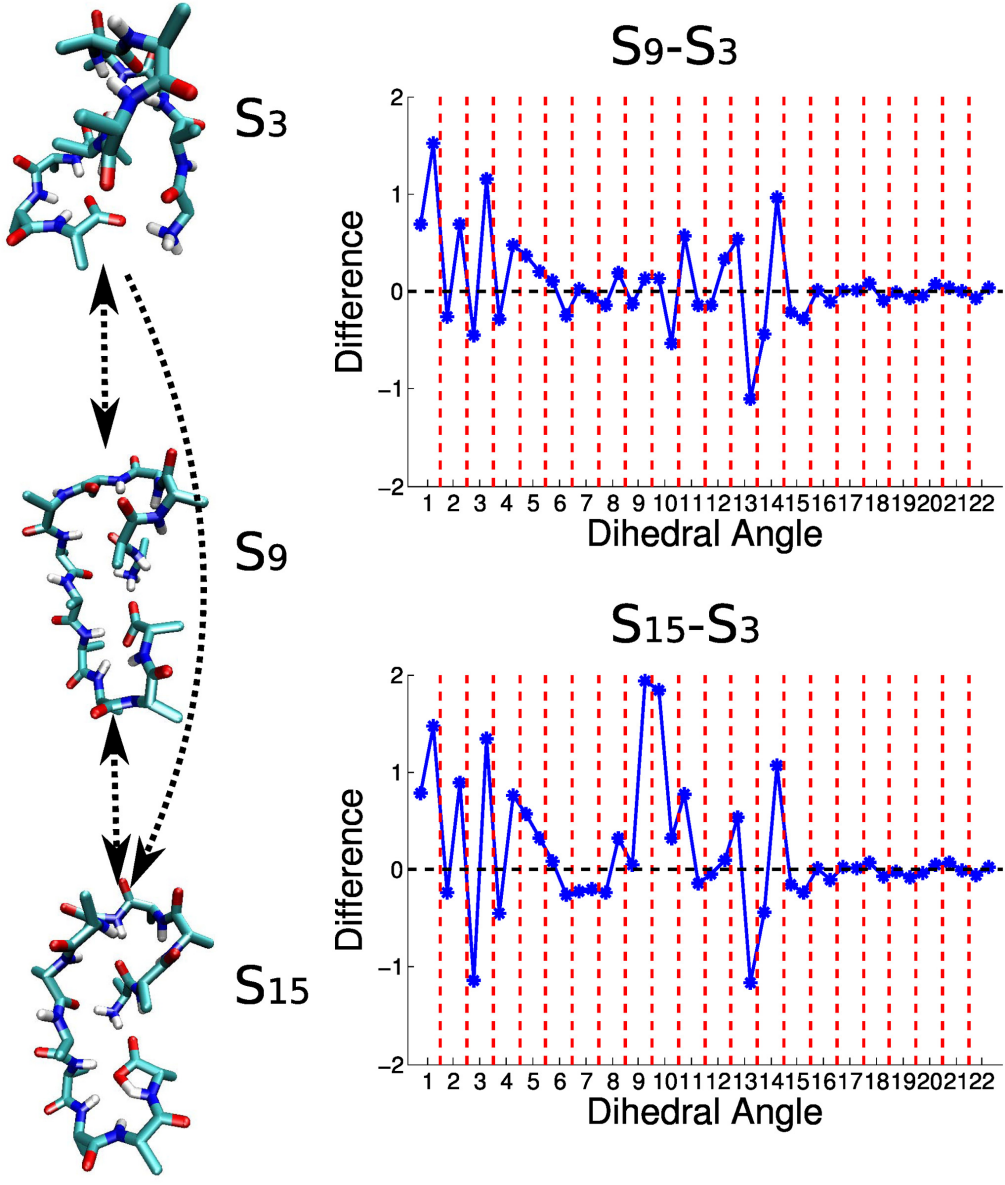
The difference graph for characterizing transition dynamics. The left panel shows the representative structures of states *S*_3_, *S*_9_ and *S*_15_ and the transition relation between these states. The difference graphs in the right panel illustrate the conformational transition between *S*_3_ and *S*_9_ (upper), as well as between *S*_3_ and *S*_15_ (lower). As introduced in the main text, the conformations in a metastable state can be characterized by a vector. The elements of the vector are the sine and cosine values of backbone dihedral angles averaged among the conformations in that state. In each difference graph, the horizontal axis marks the 22 backbone dihedral angles of Ala_12_. The *S*_3_−*S*_9_ graph shows the vector of *S*_3_ minus the vector of *S*_9_. The *S*_3_−*S*_15_ graph shows the vector of *S*_3_ minus the vector of *S*_15_.

For example, we analyze the three states *S*_3_, *S*_9_ and *S*_15_ as a transition cycle in the transition network (see Fig 6). As shown in Fig 10, upper left panel, the transition between *S*_3_ and *S*_9_ is mainly induced by the twisting of the N-terminal backbone dihedral angles 1*N*−1*C*_*α*_−1*C*′−2*N*, 1*C*′−2*N*−2*C*_*α*_−2*C*′ and 2*N*−2*C*_*α*_−2*C*′−3*N*, as well as the minor adjustment in the middle of the chain. (Here the name of an atom is composed of two parts, the integer number indicates the residue number, and the letters concretely provide the identity of the atom in a residue. Here *N* corresponds to the backbone nitrogen atom, *C*_*α*_ corresponds to the *α* carbon atom, *C*′ corresponds to the carboxyl carbon atom.) When comparing *S*_3_ and *S*_15_ (see Fig 10, lower left panel), we found that the difference between *S*_3_ and *S*_9_ is still preserved, and there is additional major difference at dihedral angles 5*N*−5*C*_*α*_−5*C*′−6*N* and 5*C*′−6*N*−6*C*_*α*_−6*C*′. This result suggests that the transition between *S*_9_ and *S*_15_ is only induced by the local adjustment of these two dihedral angles, which explains the fast transition between the two states shown in Fig 2(b). Meanwhile, the transition between *S*_3_ and *S*_9_ as well as that between *S*_3_ and *S*_15_ are more likely to be induced by the collective motion of the whole system. Usually it is hard to figure out the dynamic modes of a complex system by direct visual inspection. The difference graph shown in Fig 10 makes the dynamic modes directly observable, no matter these modes are localized or collective.

## Discussion

The trajectory mapping (TM) method and its systematical implementation developed here has wide applicability to data mining of all varieties. The metastable states of bio-molecules as well as their hierarchical organization can be systematically extracted from simulation data. Applying the TM in the long simulation data of alanine-dodeca-peptide, 28 metastable states with various life time and equilibration time were identified. These heterogeneous states could account for more than 90 percent of simulation data, which illustrates the impressive metastability of the model system.

A transition network was established and compared to the downhill folding process of protein. We found that even for this simple model system, there is considerable overlap between metastable states along the commonly used reaction coordinates. Therefore, simply projecting the simulation data to low-dimensional space might unavoidably introduce some artifact in kinetics. Such a finding testifies again the superiority of the transition network representation of bio-molecules. Since the TM also provides the local equilibrium sample of states, it is also possible to figure out the dynamic modes of inter-state transitions.

Benefiting from the rapidly increasing computational power, people are collecting massive detailed simulation data of bio-molecules. Careful analysis of these data can provide a lot of insightful information about the organization style of bio-molecules, which may greatly facilitate the rational engineering of life materials. The transition network representation has been designed to coarse-grain the dynamics of complex bio-molecules, and some methods have been subsequently invented to establish the network from high-dimensional simulation data. Since the temporal information of analytical basis functions is incorporated in the TM, we can directly find the local equilibrium sample of metastable states, which on the one hand facilitates further usage, and on the other hand ensures that the identified states are physically meaningful. The implementation of TM is flexible. It allows researchers to focus on part of the system by only selecting basis functions related to the interesting region.

Applying the TM to existing massive simulation data of proteins is currently ongoing. We would like to mention that although we only focus on the simulation data in this paper, theoretically the SMF data can also be analyzed by the TM. Recently, there have been attempts to simultaneously measure multiple intra-molecular distances in SMF experiment. TM is especially appropriate for handling such kind of multiple-dimensional data.

## Supporting Information

S1 Fig The illustration of the current clustering algorithm in TM. The trajectory-mapped vectors of an imaginary three-state system are projected to a two-dimensional space. In clustering process, the points in white region will be considered for further clustering, and the ones in shaded region have already been analyzed and will not be considered further.
(TIF)

S2 Fig The illustration of the hierarchical analysis scheme. The left panel of (a), (b) and (c) show the state structure in conformational space at three different levels. The size of a state is determined by its *τ*_*life*_. The transition relation between states is plotted with dotted arrows. The right panel of (a), (b) and (c) show the inter-state transition curve at three different levels. ‘O’ denotes the non-identified regions in simulation trajectory. (d) shows the final picture of the conformational space after identifying the sub-states of *S*_1_ and *S*_2_.
(TIF)

S3 Fig A representative conformation of Ala_12_ (a) and the orthogonality (SIP) between 4 μ s-length simulation trajectories (b). Shown are the SIP values without absolute-value manipulation.
(TIF)

S4 Fig The orthogonality (SIP) between identified metastable states in Ala_12_. The states are found respectively at three levels, 200ns, 20ns and 2ns. (a), (c) and (e) show the SIP values between states found in the same level. (b), (d), (f) show the SIP values between states found in different levels. Shown are the SIP values without absolute-value manipulation.
(TIF)

S5 Fig The 28 state-indicator curves along the 1st 4 μ s-length trajectory. (TIF)

S6 Fig The 28 state-indicator curves along the 2nd 4 μ s-length trajectory. (TIF)

S7 Fig The 28 state-indicator curves along the 4th 4 μ s-length trajectory. (TIF)

S8 Fig The 28 state-indicator curves along the 5th 4 μ s-length trajectory. (TIF)

S9 Fig The state-indicator curves of the sub-states of *S*_1_ in *Ala*_12_ along the 2nd 4 μ s-length trajectory. The upper panel shows the full curves. The lower panel shows the enlarged view of the region from 3.1 μ s to 3.3 μ s.
(TIF)

S10 Fig The state-indicator curves of the sub-states of *S*_2_ in *Ala*_12_ along the 2nd 4 μ s-length trajectory. The upper panel shows the full curves. The lower panel shows the enlarged view of the region from 0.0 μ s to 0.2 μ s.
(TIF)

S11 Fig The representative structures of the sub-states of *S*_1_ and their inter-relation. The left panel shows the representative structures, the right panel shows the difference graphs between sub-states. The shown graphs are selected to reflect the most localized differences between the sub-states.
(TIF)

S12 Fig The representative structures of the sub-states of *S*_2_ and their inter-relation. The left panel shows the representative structures, the right panel shows the difference graphs between sub-states. The shown graphs are selected to reflect the most localized differences between the sub-states.
(TIF)

S1 Table The number of trajectory pieces defining the metastable states and the average SIP values. (PDF)

S2 Table The proportion of data accountable by the identified metastable states. At certain time point, the simulation trajectory is considered as accountable by the identified metastable state only if the summation of state-indicator curves at this time point is larger than 0.9.
(PDF)

S1 Text Details of clustering algorithm. (PDF)
