# Supplementary figures and images for: Systematically Constructing Kinetic Transition Network in Polypeptide from Top to Down: Trajectory Mapping
Source: PLoS One. 2015 May 11;10(5):e0125932. doi: 10.1371/journal.pone.0125932 (PMC4427365; doi:10.1371/journal.pone.0125932)

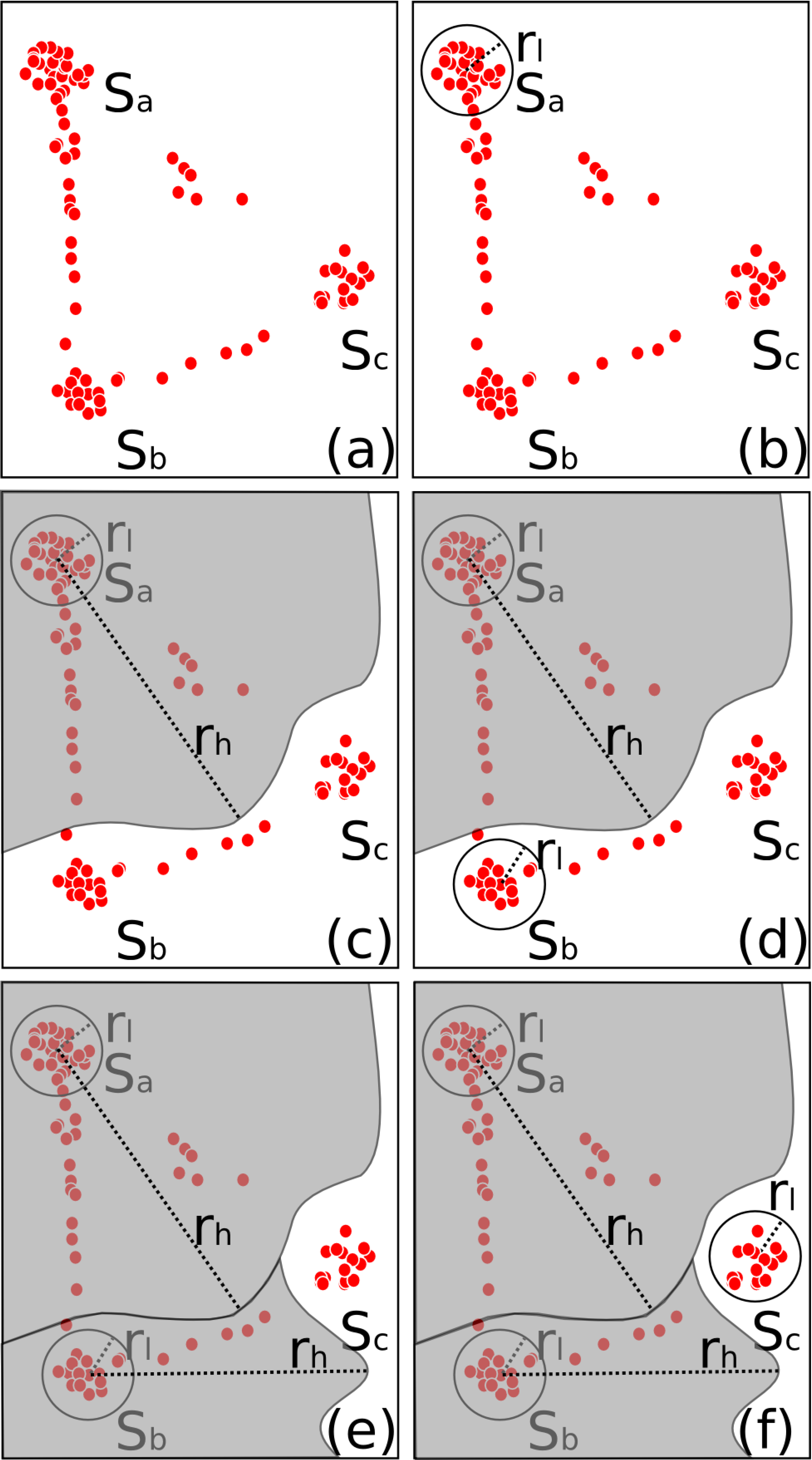

Supplement: S1 Fig — The trajectory-mapped vectors of an imaginary three-state system are projected to a two-dimensional space. In clustering process, the points in white region will be considered for further clustering, and the ones in shaded region have already been analyzed and will not be considered further. (TIF) [file pone.0125932.s001.tif]

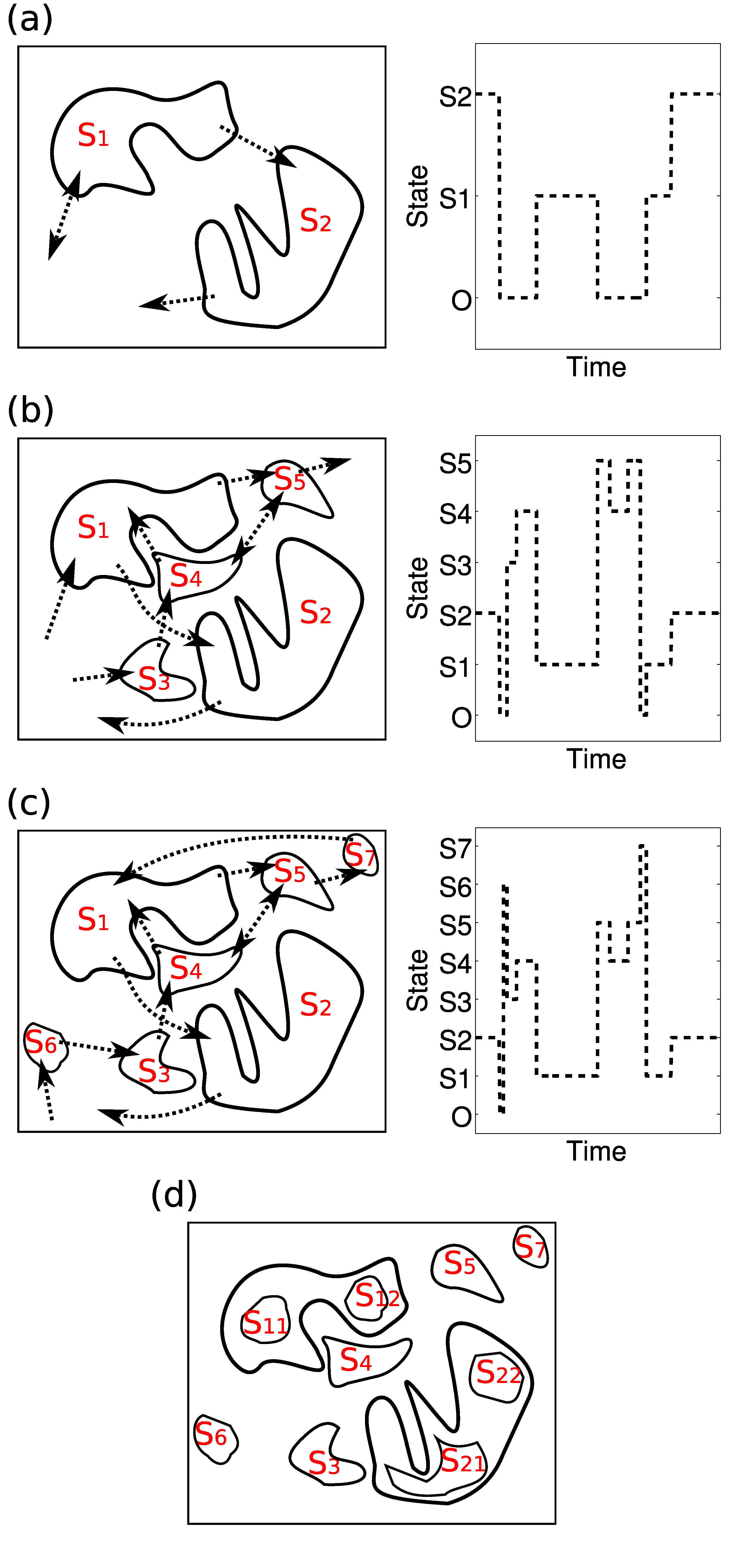

Supplement: S2 Fig — The left panel of (a), (b) and (c) show the state structure in conformational space at three different levels. The size of a state is determined by its τ life. The transition relation between states is plotted with dotted arrows. The right panel of (a), (b) and (c) show the inter-state transition curve at three different levels. ‘O’ denotes the non-identified regions in simulation trajectory. (d) shows the final picture of the conformational space after identifying the sub-states of S 1 and S 2. (TIF) [file pone.0125932.s002.tif]

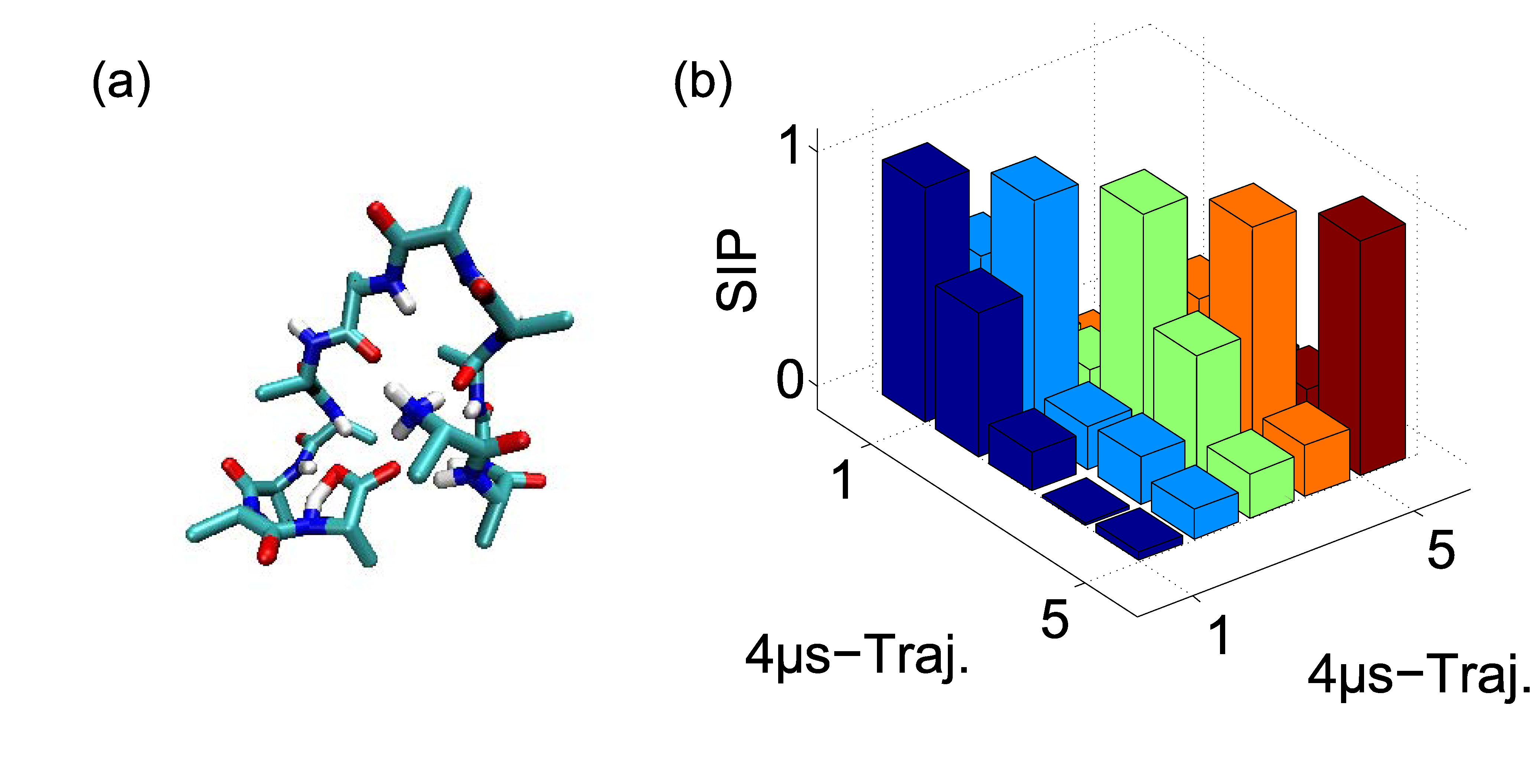

Supplement: S3 Fig — Shown are the SIP values without absolute-value manipulation. (TIF) [file pone.0125932.s003.tif]

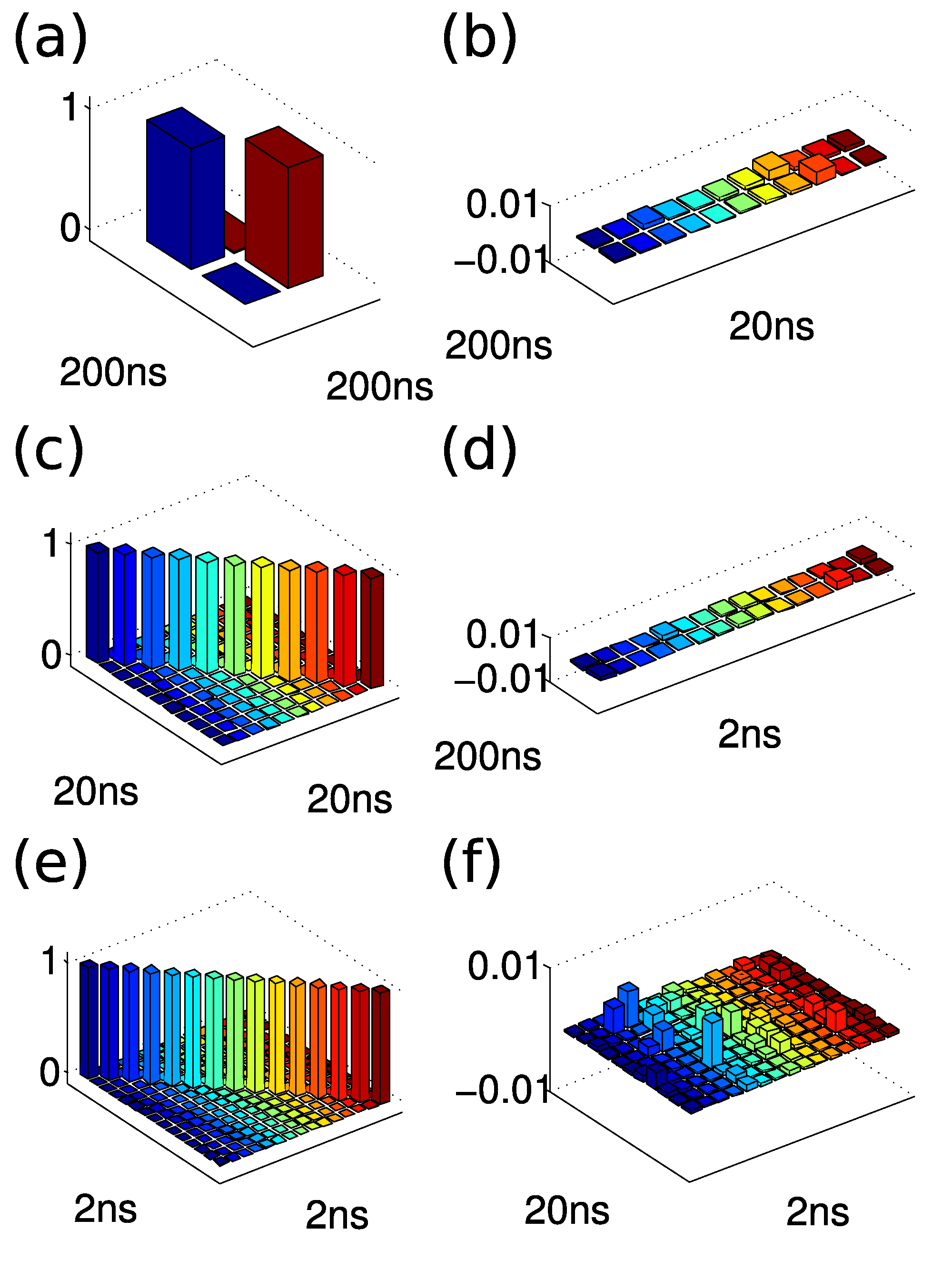

Supplement: S4 Fig — The states are found respectively at three levels, 200ns, 20ns and 2ns. (a), (c) and (e) show the SIP values between states found in the same level. (b), (d), (f) show the SIP values between states found in different levels. Shown are the SIP values without absolute-value manipulation. (TIF) [file pone.0125932.s004.tif]

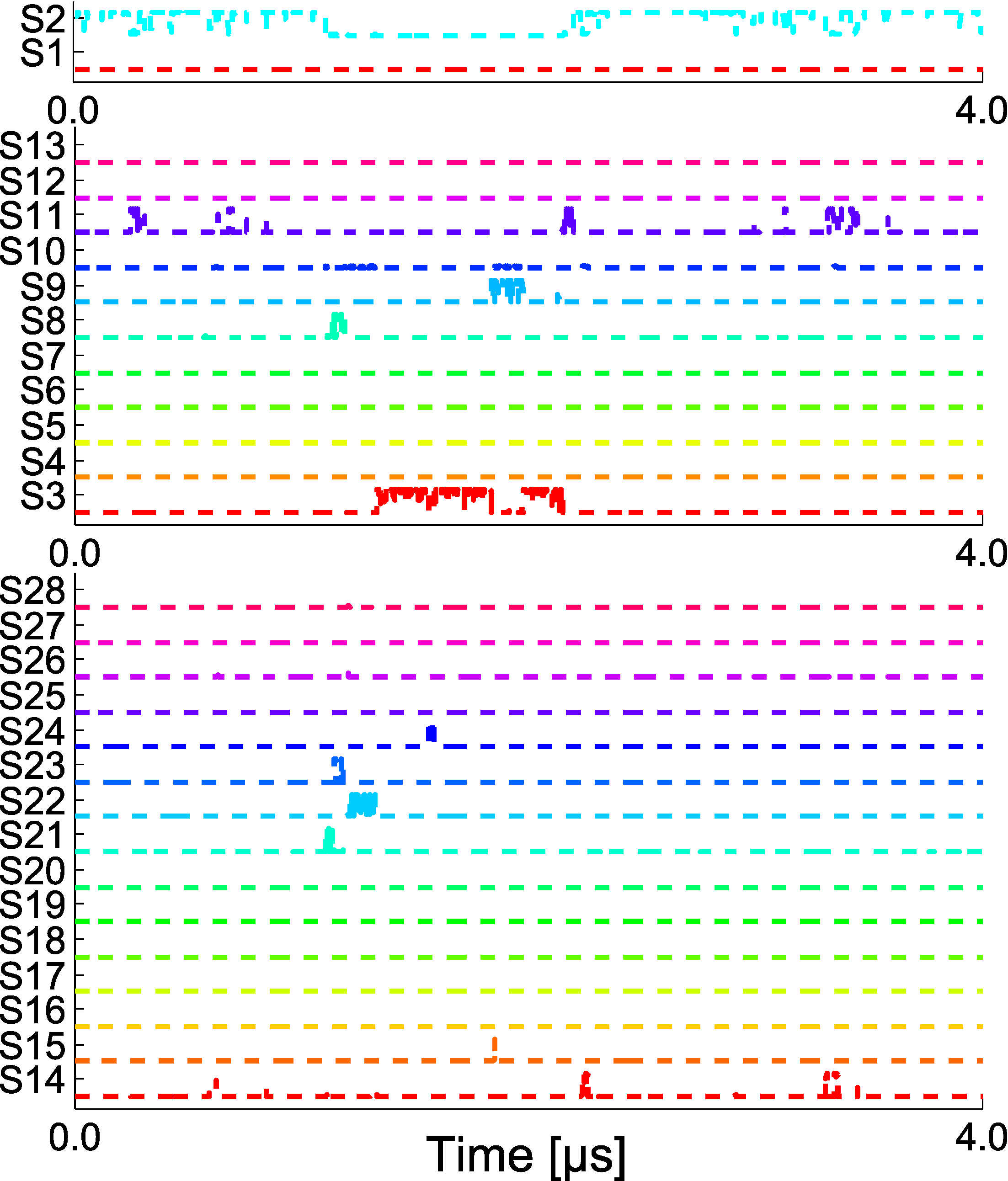

Supplement: S5 Fig — (TIF) [file pone.0125932.s005.tif]

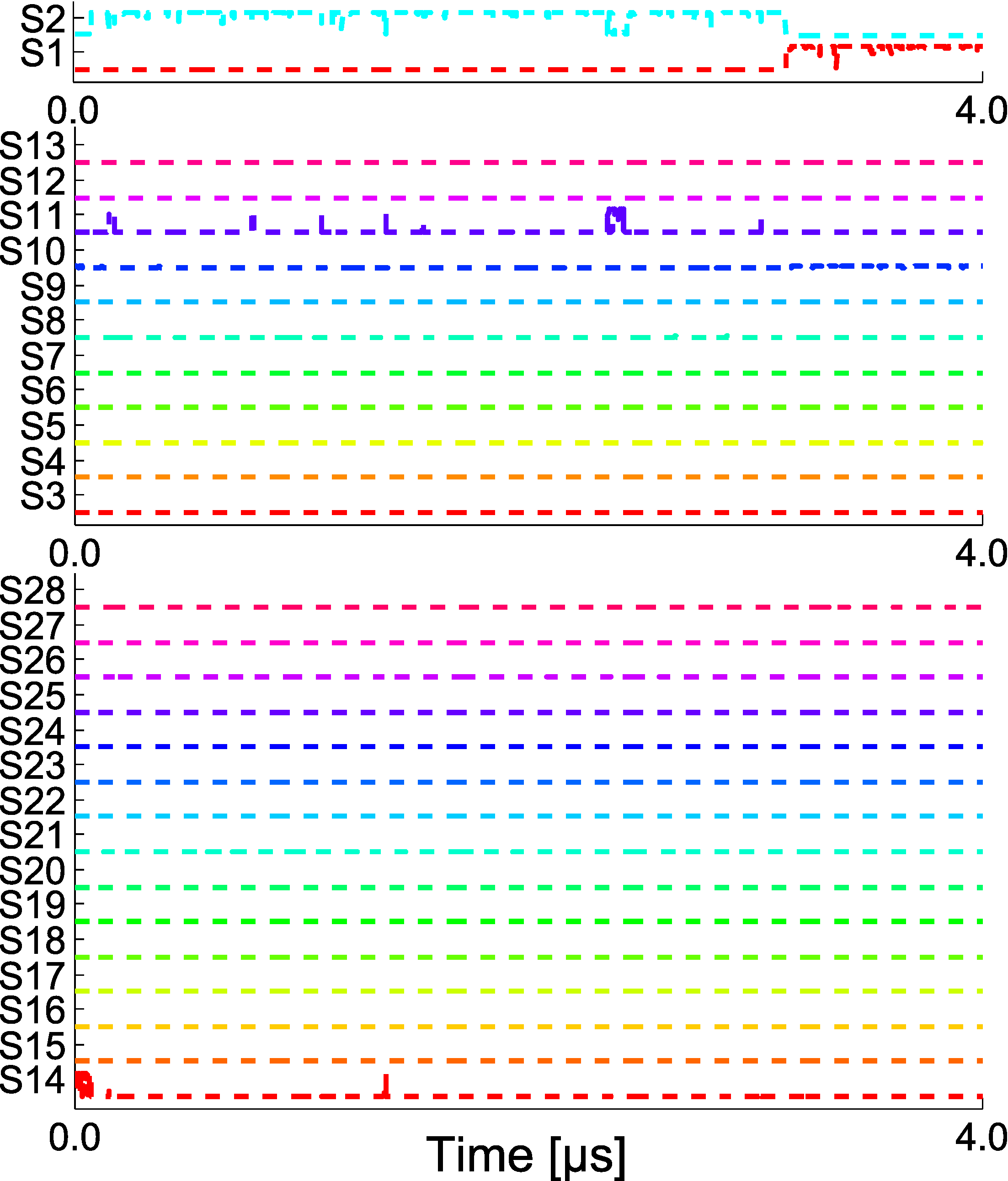

Supplement: S6 Fig — (TIF) [file pone.0125932.s006.tif]

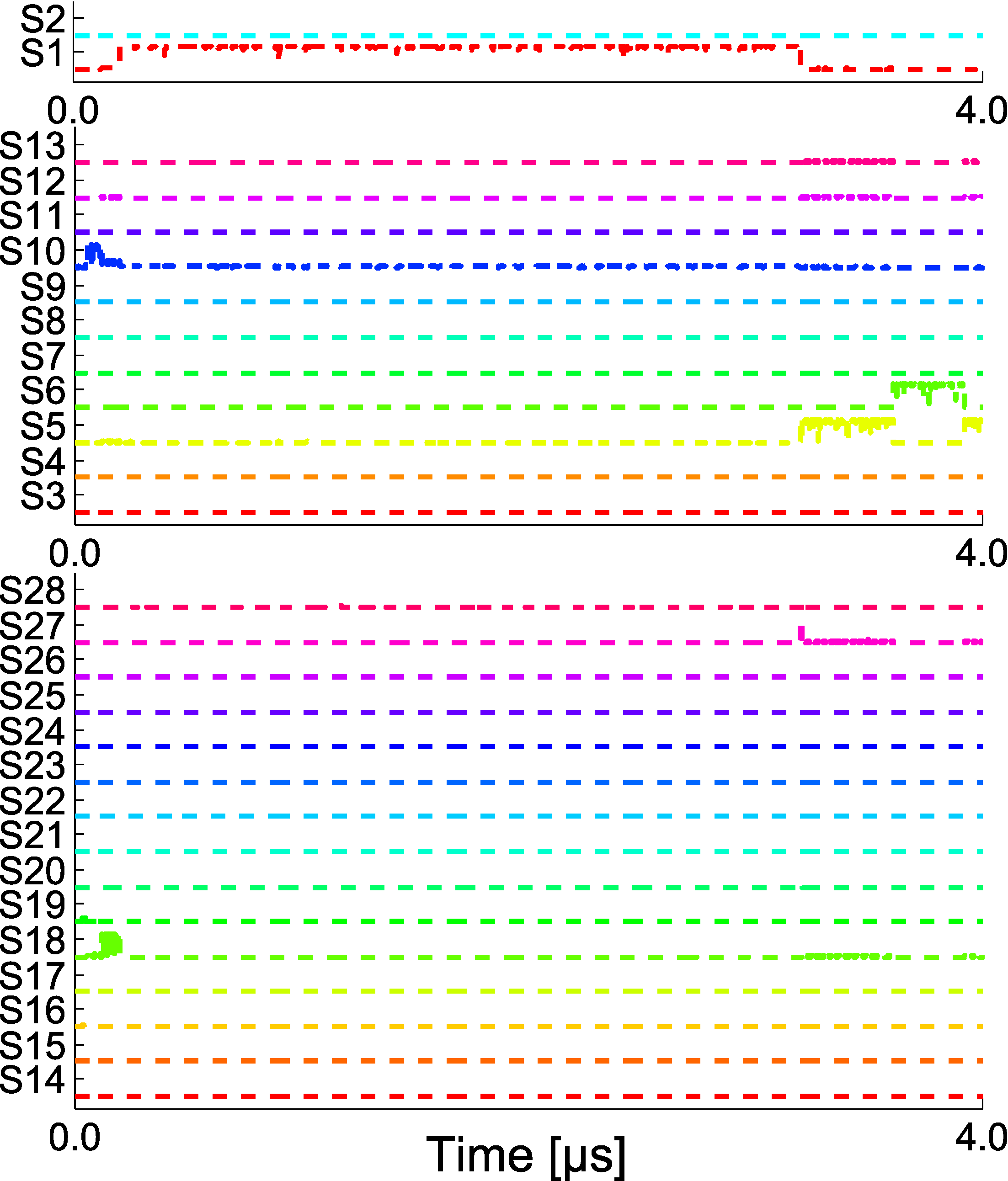

Supplement: S7 Fig — (TIF) [file pone.0125932.s007.tif]

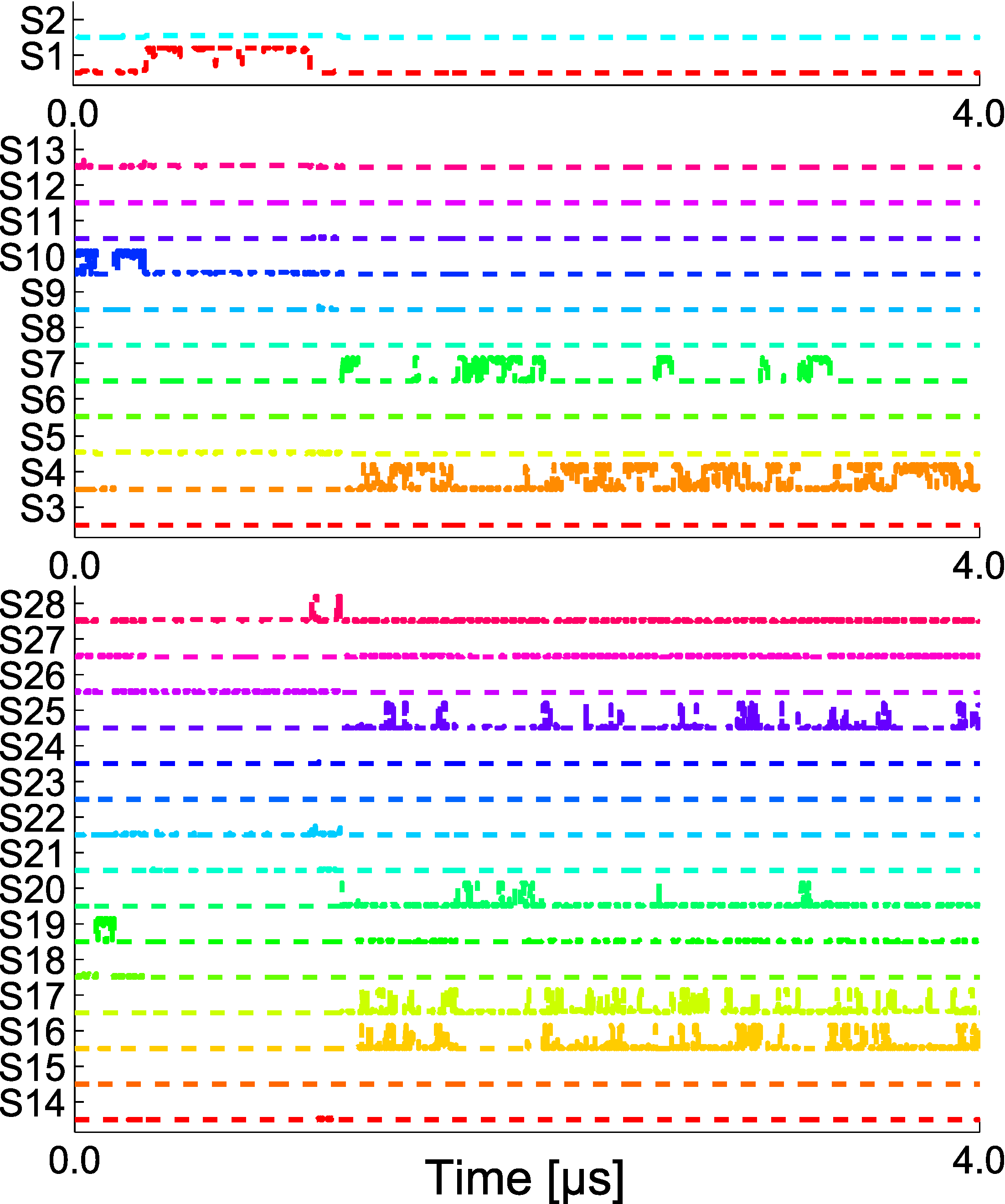

Supplement: S8 Fig — (TIF) [file pone.0125932.s008.tif]

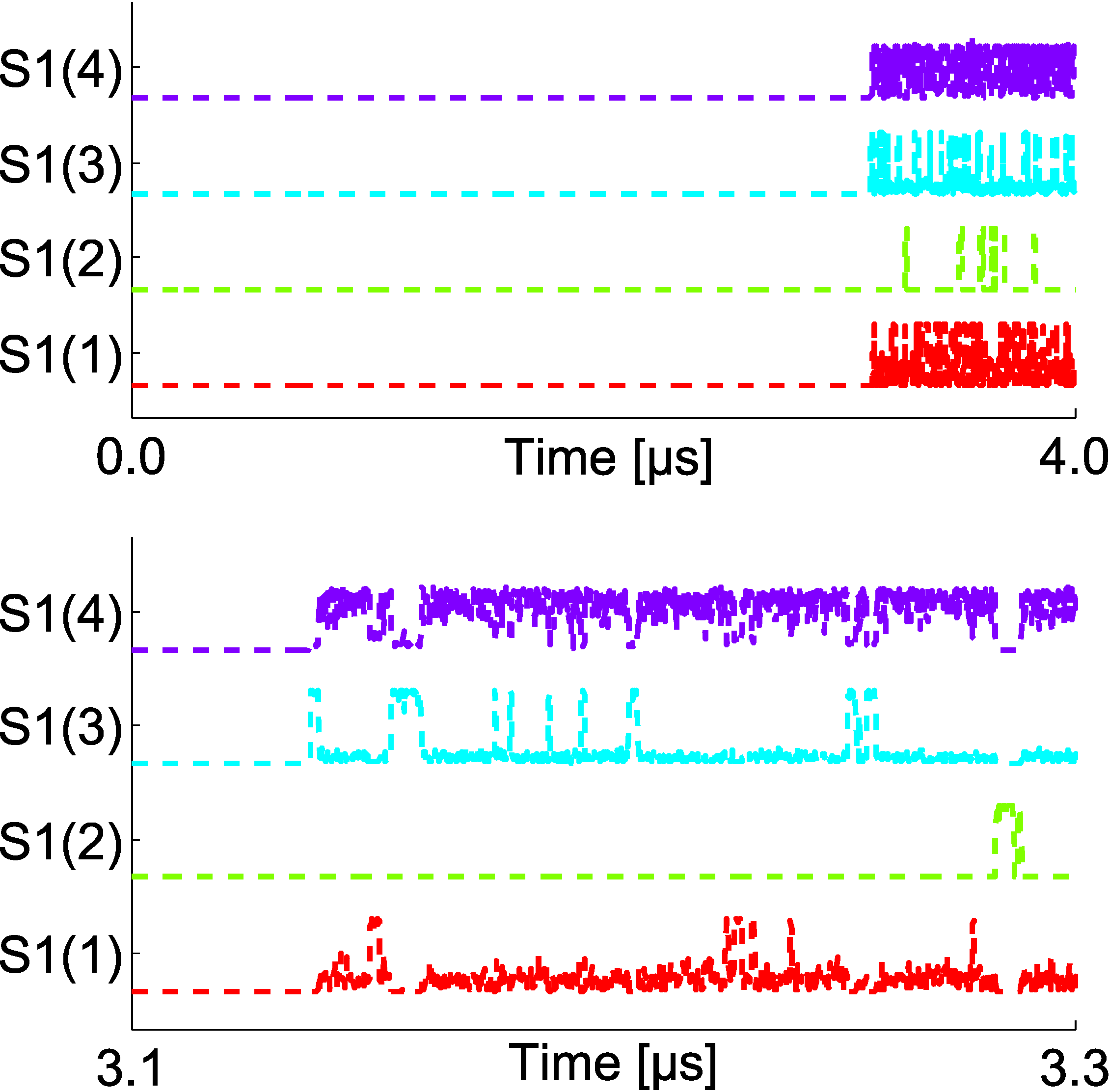

Supplement: S9 Fig — The upper panel shows the full curves. The lower panel shows the enlarged view of the region from 3.1 μ s to 3.3 μ s. (TIF) [file pone.0125932.s009.tif]

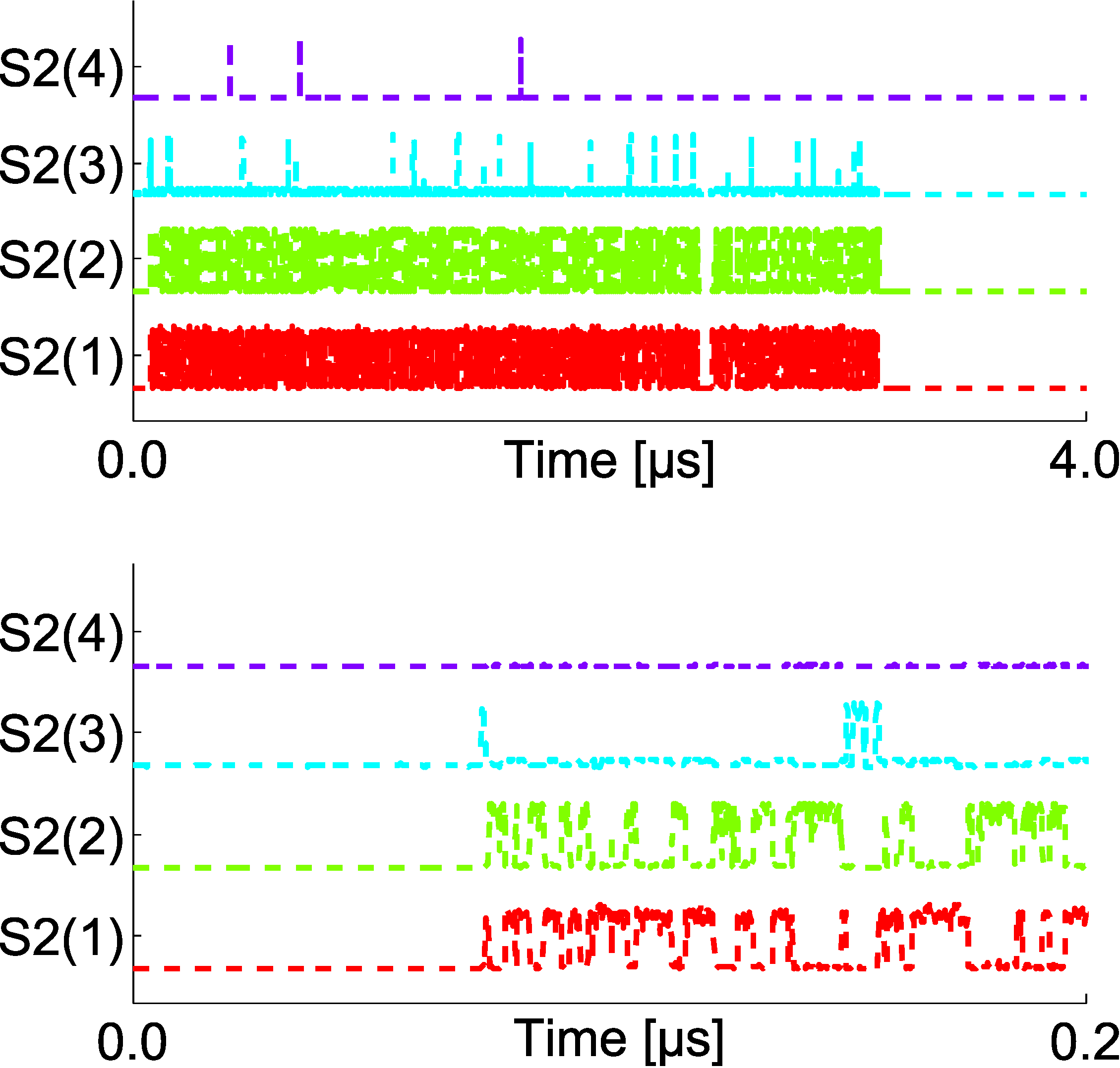

Supplement: S10 Fig — The upper panel shows the full curves. The lower panel shows the enlarged view of the region from 0.0 μ s to 0.2 μ s. (TIF) [file pone.0125932.s010.tif]

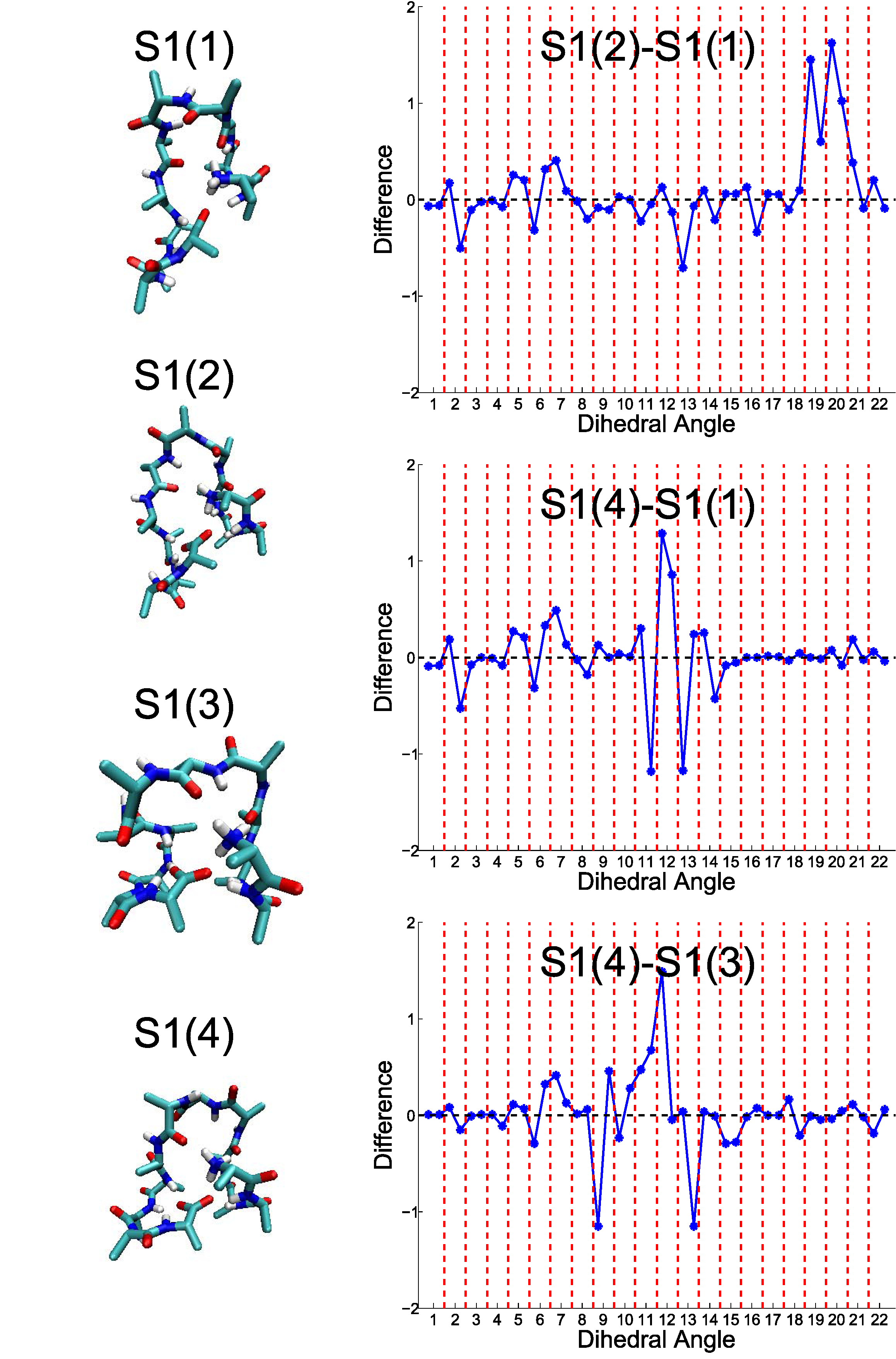

Supplement: S11 Fig — The left panel shows the representative structures, the right panel shows the difference graphs between sub-states. The shown graphs are selected to reflect the most localized differences between the sub-states. (TIF) [file pone.0125932.s011.tif]

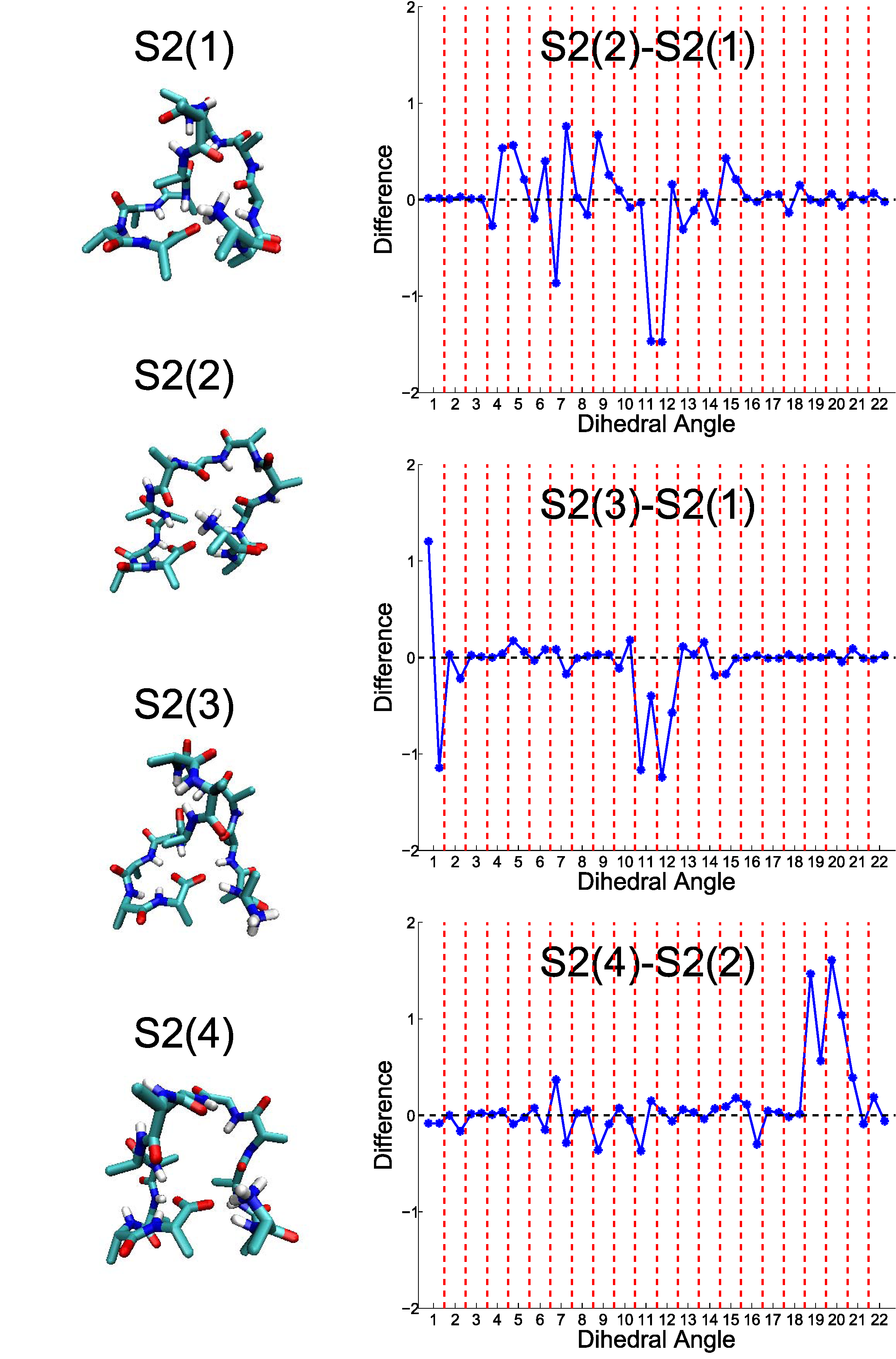

Supplement: S12 Fig — The left panel shows the representative structures, the right panel shows the difference graphs between sub-states. The shown graphs are selected to reflect the most localized differences between the sub-states. (TIF) [file pone.0125932.s012.tif]
