# Supplementary material for: Systematically Constructing Kinetic Transition Network in Polypeptide from Top to Down: Trajectory Mapping
Source: PLoS One. 2015 May 11;10(5):e0125932. doi: 10.1371/journal.pone.0125932 (PMC4427365; doi:10.1371/journal.pone.0125932)
Supplement: S1 Table — (PDF) [file pone.0125932.s013.pdf]

States	Before Refining	Average SIP	After Refining	Average SIP
$\tau = 200\text{ns}$				
S_1	26	0.9686	26	0.9675
S_2	21	0.9633	11	0.9817
$\tau = 20\text{ns}$				
S_3	58	0.9944	45	0.9957
S_4	31	0.9685	7	0.9827
S_5	24	0.9783	3	0.9932
S_6	15	0.9888	14	0.9889
S_7	14	0.9850	5	0.9842
S_8	11	0.9705	2	0.9962
S_9	8	0.9716	3	0.9933
S_{10}	7	0.9611	1	1
S_{11}	5	0.9799	3	0.9906
S_{12}	5	0.9922	5	0.9921
S_{13}	5	0.9642	3	0.9760
$\tau = 2\text{ns}$				
S_{14}	42	0.9762	9	0.9799
S_{15}	34	0.9781	15	0.9835
S_{16}	16	0.9711	2	0.9899
S_{17}	25	0.9475	2	0.9792
S_{18}	28	0.9589	2	0.9875
S_{19}	22	0.9593	4	0.9717
S_{20}	20	0.9469	2	0.9841
S_{21}	18	0.9646	7	0.9825
S_{22}	20	0.9522	1	1
S_{23}	14	0.9740	9	0.9761
S_{24}	13	0.9628	8	0.9656
S_{25}	7	0.9530	2	0.9886
S_{26}	10	0.9439	2	0.9882
S_{27}	5	0.9723	4	0.9753
S_{28}	5	0.9885	2	0.9942
