# Supplementary material for: Systematically Constructing Kinetic Transition Network in Polypeptide from Top to Down: Trajectory Mapping
Source: PLoS One. 2015 May 11;10(5):e0125932. doi: 10.1371/journal.pone.0125932 (PMC4427365; doi:10.1371/journal.pone.0125932)
Supplement: S2 Table — At certain time point, the simulation trajectory is considered as accountable by the identified metastable state only if the summation of state-indicator curves at this time point is larger than 0.9. (PDF) [file pone.0125932.s014.pdf]

Trajectory	1	2	3	4	5	Average
Proportion(%)	93.21	96.51	91.01	96.05	76.70	90.70
