# Supplementary material for: Systematically Constructing Kinetic Transition Network in Polypeptide from Top to Down: Trajectory Mapping
Source: PLoS One. 2015 May 11;10(5):e0125932. doi: 10.1371/journal.pone.0125932 (PMC4427365; doi:10.1371/journal.pone.0125932)
Supplement: S1 Text — (PDF) [file pone.0125932.s015.pdf]

Details of clustering algorithm

We list the details of applied clustering algorithm including some tricks.

1. For each trajectory-mapped vector $\vec{v}_k$, calculate its SIP with the other vectors. Record the total number N_{ne}^k of the vectors that have their SIP with $\vec{v}_k$ larger than r_l. if $\max_k N_{ne}^k > N_{ne}^{std}$, a new metastable state is found.

2. To refine the group of vectors found in 1, we calculate the average vector of them and the SIP between each vector and the average one. A vector is kept inside the group only if the SIP between it and the average vector is larger than r_l. This procedure is repeated until the composition of the group of vectors becomes stable.

3. For each vector out of the refined group of vectors determined in 2, its SIP with the average vector of the refined group is calculated. A vector is retained for the following analysis only if the SIP is smaller than r_h, which is set as 0.1 in the paper. By doing so, we collect another group of vectors that are almost orthogonal to the vectors defining the new metastable state, the other vectors are omitted in the following clustering process.

4. If the total number of the remaining vectors in 3 is larger than N_{ne}^{std}, 1 to 3 are repeated to search for the other metastable states. Otherwise, the process is ended.

The identified metastable states can be further refined. For each metastable state, we calculate the representative vector by averaging the vectors defining the state, then the SIP between representative vectors of states as the interstate SIP. We demand the interstate SIP is smaller than r_s, is set as 0.01 in the work. If a pair of states does not meet this demand, we can adjust them as follows. For each vector in one of the two states, we calculate its SIP with the representative vector of another state. The vector with the largest above-defined SIP value in the two states is deleted. The SIP-calculation and vector-deletion processes are repeated until the r_s-standard is fulfilled.
